# Structural Characterization of Peripolin and Study of Antioxidant Activity of HMG Flavonoids from Bergamot Fruit

**DOI:** 10.3390/antiox11101847

**Published:** 2022-09-20

**Authors:** Lucia Bartella, Fabio Mazzotti, Ines Rosita Talarico, Giuseppina De Luca, Ilaria Santoro, Mario Prejanò, Costanza Riccioni, Tiziana Marino, Leonardo Di Donna

**Affiliations:** 1 QUASIORA Laboratory, AGRINFRA Research Net, Università Della Calabria, Via P. Bucci Cubo 12/D, I-87036 Arcavacata di Rende, CS, Italy; 2 Dipartimento di Chimica e Tecnologie Chimiche, Università Della Calabria, Via P. Bucci, Cubo 12/D, I-87030 Rende, CS, Italy; 3 Esserre Pharma SrL R&D Department, Via Flaminia Nuova 260, I-00191 Roma, RM, Italy

**Keywords:** bergamot, citrus bergamia, high-resolution mass spectrometry, structure elucidation, HMG flavonoids, statin, peripolin, antioxidant activity

## Abstract

The structural characterization of a new flavonoid from bergamot fruit (Citrus bergamia Risso) carrying the 3-hydroxy-3-methyl glutaryl (HMG) ester moiety has been accomplished, and its antioxidant ability was tested from a chemical point of view. The peculiarity of the new molecule, named peripolin, relies on the presence of the HMG chemical group linked to the sugar portion of neoeriocitrin; the structure was elucidated using both high-resolution mass spectrometry and nuclear magnetic resonance experiments performed on the purified molecule extracted from the fruit. The antioxidant ability of the new molecule was tested by classical chemical approaches, such as DPPH, ABTS and FRAP assays, and from a theoretical point of view. ^1^H and ^13^C NMR experiments and HR-ESI-MS/MS experiments show unequivocally that the HMG moiety is linked to the primary position of the glucose unit of neohesperidose, while the chemical tests and the computational results show that peripolin possesses strong antioxidant behavior, similar to that of neoeriocitrin and remarkably higher respect to the other flavonoids present in the fruit. Furthermore, the quantitative assays carried out by UPLC-MS/MS showed that its amount in the fruit is similar to that of the other main flavonoids. Furthermore, molecular dynamics simulations allowed us to investigate the possible conformations adopted by the antioxidants in the presence of water molecules. In particular, the switch of open-closed conformations of HMG-containing species was evidenced. As far as the reaction with DPPH, the calculation of ΔG_rea_ supported the experimental outcomes regarding the peripolin and neoeriocitrin activity. In conclusion, bergamot fruit, already known for its potential to lower the level of blood cholesterol, has been proven to contain molecules such as neoeriocitrin and the newly characterized peripolin, which could have important in-vivo antioxidant characteristics.

## 1. Introduction

**Figure 1:**
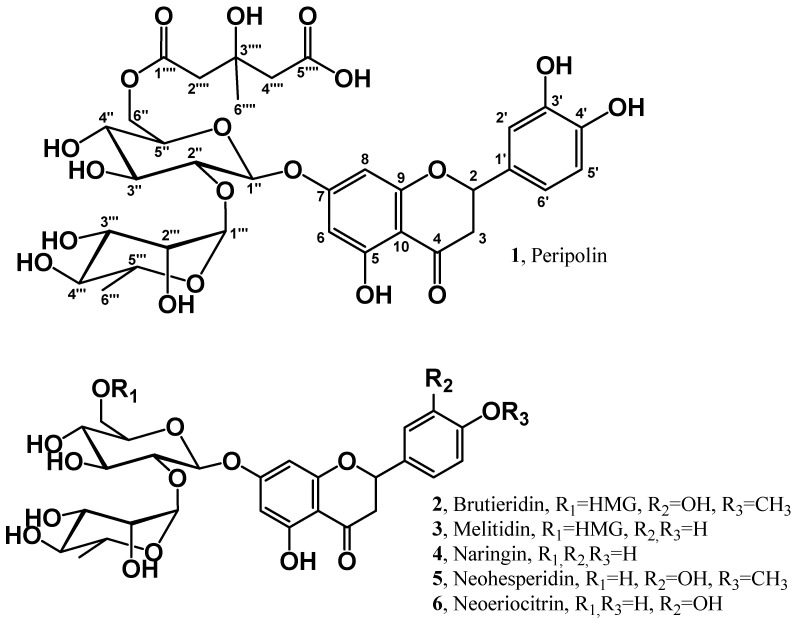
Structural formula of Peripolin and the main flavonoids from bergamot.

Citrus fruits are among the most extensively grown fruit tree crops in the world. The derived juices, for example, represent a vast globally traded commodity. It is estimated that more than 143 million tons of citrus fruits are produced every year in the world [1]. Bergamot (Citrus bergamia Risso) represents only a negligible part of citrus production (20,000 tons) since its crop is limited for most to the area of Reggio Calabria in southern Italy [2]; it is used primarily to extract the essential oil present in the peel of the fruit (epicarp). For decades, the inner part of the fruit (i.e., the juice vesicles and the albedo) was considered no more than waste, but recently, many papers reported the presence of valuable phytochemicals and nutraceuticals [3]. The juice, for example, is particularly rich in flavonoids [4], a class of polyhydroxy aromatics (polyphenols), which are known to possess several physiological functions related to their antioxidant [5], antiviral [6], anti-thrombotic [7], and anticarcinogenic [8] activities. Naringin (**4**, Figure 1), neohesperidin (**5**) and neoeriocitrin (**6**) are the major components of the polyphenolic fraction; C-glycosylated flavanone and flavone are less abundant [9]. Recently, it has been reported that the juice possesses hypocholesterolemic activity [10,11] that may be ascribed to two statin-like flavanones (**2**,**3**, Figure 1) discovered in the bergamot fruit [12,13,14] and present in considerable amounts amount. Their peculiarity relies on the presence of the 3-hydroxy-3-methylglutaryl ester moiety (HMG), which makes these molecules more soluble in water and, in principle, more readily absorbable after oral ingestion [15,16]. 

The discovery of the beneficial activity of the flavonoid extracts from the pulp led to the development of new products such as, for example, nutraceuticals (capsules, pills or soluble granular powders). The identification and characterization of vegetable microcomponents are of major importance either for the authentication of botanicals, especially if they are used as food additives [17], or to gain information about the authenticity of food products [18]. 

The present work describes the full structural characterization performed by high-resolution nuclear magnetic resonance (HR-NMR) and high-resolution tandem mass spectrometry (HR-ESI-MS/MS) of peripolin (**1**, eriodictyol 7-(2″-α-rhamnosyl-6″-(3⁗-hydroxy-3⁗-methylglutaryl)-β-glucoside), Figure 1) a new HMG derivative in bergamot detected previously, but whose chemical structure was never established [19]. The purified molecule was used as an authentic standard to estimate the amount of the new compound in bergamot-based juices and nutraceutical ingredients. In addition, the antioxidant characteristics of pure flavonoids (**1**–**3**) and their HMG counterpart, e.g., Brutieridin, Melitidin and Peripolin was investigated from a theoretical point of view and by experimental assays.

## 2. Materials and Methods

### 2.1. Chemicals, Standards and Bergamot Samples

HPLC grade solvents, reagents and pure flavonoids (**4**–**6**) were purchased from Merck (Milano, Italy); bergamot juices and fruits were provided by Azienda Pratticò (Africo Nuovo, Italy); standard purified brutieridin and melitidin were obtained in-house [20]; bergamot powder extract was provided by Esserre Pharma SrL (Rome, Italy).

### 2.2. Purification and Characterization of Peripolin

#### 2.2.1. Sample Preparation

Fruits of bergamot were collected in December 2021 and then stored at −20° C. The fruits were squeezed to separate the juice from the rest of the pulp; the latter was extracted with water (3 × 210) mL at 20 °C for 1 h. The extracted solution and the juice were filtered, brought together again and then passed through a C_18_ cartridge (10 g, Supelco Inc., Bellefonte, PA, USA) in 50 mL aliquots. The loaded stationary phase was initially washed with water (2 × 50 mL) to remove the sugars and the polar acidic fraction, then eluted with 50 mL of methanol to collect the flavonoid fraction. Each aliquot passed through the resin provided, once evaporated under rotary vacuum, ca. 100 mg of raw flavonoid fraction, for a total of 1.5 g. The latter amount was submitted to the fractionation procedure.

#### 2.2.2. Fractionation and Purification of Peripolin by Semipreparative HPLC-UV

The purification step was performed using a Fractionlynx semi-preparative HPLC system (Waters Corp., Milford, MA, USA) working in semipreparative mode; the system was composed of an autosampler/collector Waters 2767 Sample Manager, a 2535 preparative pump, a 2489 UV detector and a 515 make-up pump. The column used was a 100 × 21 mm C_18_ Luna from Phenomenex (Torrance, CA, USA). The purification consisted of two steps: in the first step 400 μL of a solution, prepared to dissolve 100 mg of raw flavonoid fraction in 1 mL, were injected into the semipreparative system and fractionated by means of an isocratic run performed using H_2_O/MeOH (60/40) as eluents at 21 mL/min flow rate; the run time was 14 min, and the UV detector was set at 280 nm. The collected fractions (4.2 mL each) were analyzed by HPLC-UV and mass spectrometry (see below) to identify those containing partially purified peripolin; the latter were brought together, evaporated under vacuum and then lyophilized. The second purification step was performed by injecting 400 μL of a solution prepared to dissolve 40 mg of the lyophilized fraction in 1 mL under the same conditions as the first purification step. The fractions (2 mL each) were analyzed again by HPLC-UV and mass spectrometry in order to assess the purity.

#### 2.2.3. HPLC/UV-MS Analysis of Fractions and Tissues Extracts

HPLC/UV and mass spectrometry evaluated the composition of each fraction from the semipreparative HPLC experiments. The system used was the Fractionlynx working in analytical mode. The separation was performed using a 250 × 4.6 mm, 5 μm reversed-phase C_18_ Luna-Phenomenex column at a flow rate of 1 mL/min. The run time was 70 min, and the mobile phase was composed of 0.1% formic acid in water (solvent A) and methanol (solvent B). The chromatographic run (90 min) consisted of the following steps: isocratic at 80% A for 7 min; linear gradient from 80% A to 70% A in 10 min; isocratic at 70% A for 5 min; linear gradient from 70% A to 60% A in 10 min; isocratic at 60% A for 5 min; linear gradient from 60% A to 50% A in 10 min; isocratic at 50% A for 5 min; linear gradient from 50% A to 40% A in 5 min; isocratic at 40% A for 5 min; linear gradient from 40% A to 20% A in 10 min; isocratic at 20% A for 5 min; linear gradient from 20% A to 80% A in 5 min; equilibration of the column at 80% A for 8 min. The UV detector was set at 280 nm. The UV peaks were collected at the outlet of the UV detector and injected into the mass spectrometer.

The MS analyses were carried out using a TSQ Quantum Vantage triple-stage quadrupole mass spectrometer (Thermo Fisher Scientific, San José, CA, USA) equipped with a heated electrospray ionization (HESI II) source operating in negative ion mode by direct infusion (5 μL/min) with the following conditions: spray voltage, −3.5 kV; capillary and vaporizer temperatures, 270 and 280 °C, respectively; auxiliary and sheath gas at 46 and 40 arbitrary units (au), respectively

#### 2.2.4. High-Resolution Mass Spectrometry

The high-resolution electrospray experiments were carried out using the Exploris 120 high-resolution mass spectrometer (Thermo Fisher Scientific, San José, CA, USA) equipped with heated electrospray ionization (H-ESI II) probe and an Orbitrap analyzer; the mass spectrometer was hyphenated with a Vanquish system consisting of HPLC pump and autosampler (Thermo Fisher Scientific, San José, CA, USA). Solutions coming from the purified fractions were injected into the HPLC-HR-ESI-MS system using the following conditions: spray voltage: 3.5 kV, (−4.0 kV in negative mode); sheath gas, aux gas and sweep gas 50, 10, 1 a.u., respectively; ion transfer tube temperature: 310 °C; vaporizer temperature 320 °C; the scan range was set in the range 500–1000 *m/z*, while the RF lens was set to 70% of the maximum value and the orbitrap resolution was set 60,000. MS^2^ experiments were performed in data-dependent mode targeting the exact masses of protonated molecule [M + H]^+^ (*m/z* 741.2240) and deprotonated molecule [M − H]^−^ (*m/z* 739.2086) at 60,000 resolution. The system was calibrated externally to a maximum error of 2 ppm. The scan range was automatically set, while the collision energy was set to 10% and 30% of the maximum value in positive and negative mode, respectively. The molecular formulae were evaluated by Excalibur software (Thermo Fisher Scientific, San José, CA, USA).

#### 2.2.5. Nuclear Magnetic Resonance

^1^H and ^13^C NMR spectra were recorded at 25 °C on a Bruker Avance 500 MHz (^1^H:500.13 MHz, ^13^C:125.77 MHz) instrument (Rheinstetten, Germany) by dissolving pure samples in CD_3_OD.

#### 2.2.6. Sample Preparation for UPLC-MS/MS Assay

Bergamot beverages: An amount of 1 mL of the sample was filtered and properly diluted with internal standards. Bergamot powder extracts: Around 5 mg of extract were dissolved in H_2_O/MeOH (80/20), then properly diluted and mixed with IS.

#### 2.2.7. UPLC-MS/MS Assay

HPLC-MS/MS quantitation analysis was carried out on a system from Thermo Scientific composed of a UHPLC Accela pump coupled to a TSQ Quantum Vantage triple-stage quadrupole mass spectrometer (Thermo Fisher Scientific, San José, CA, USA). The chromatographic analysis was performed using a C_18_ reversed-phase column, Hypersil (2.1 × 50 mm, 3 μm particle size, Thermo Fisher Scientific). H_2_O (A) and MeOH (B) were used as solvents for chromatographic separation and the elution gradient was the following: isocratic at 80% A from t = 0.0 min to t = 1.46 min; gradient until 3.54 min to 70% A; isocratic at 70% A from t = 3.54 min to t = 4.58 min; gradient until 6.66 min to 50% A; isocratic at 50% A from t = 6.66 min to t = 7.77 min; gradient until 9.78 min to 40% A; isocratic 40% A from t = 9.78 min to t = 10.82 min; gradient until 11.76 min to 30% A; isocratic 30% A from t = 11.76 min to t = 12.90 min; gradient until 14.90 min to 20% A; isocratic 20% A from t = 14.90 min to t = 16.00 min; return to initial condition til 18 min; re-equilibration time 2 min. The flow rate was set at 0.3 mL/min, and the sample injection volume was 5 µL. A further switching valve located on the mass spectrometer was used to divert the LC flow to waste at the first minute and after 14.00 min of each analysis to protect the MS source from contamination. Mass spectrometry was performed, acquiring spectral data on a triple-quadrupole mass analyzer equipped with a heated electrospray ionization (HESI II) source operating in negative ion mode with the following conditions: spray voltage, −3.5 kV; capillary and vaporizer temperatures, 270 and 280 °C, respectively; auxiliary and sheath gas at 46 and 40 arbitrary units (au), respectively. Quantitative analysis was performed by multiple reaction monitoring (MRM) following specific transitions for flavonoid standards and caffeic acid that was used as an internal standard. The collision-induced dissociation gas was argon at a pressure of 1.0 mTorr, while the mass resolution at the first (Q1) and third (Q3) quadrupoles was set at 0.7 Da at full width at half-maximum (FWHM). The S-lens rf amplitude and the collision energy (CE) were optimized individually per compound. All valve positions and instrument parameters were controlled by Xcalibur software, version 2.0.0 (Thermo Fisher Scientific). The total HPLC-MS/MS method run time was 20 min.

### 2.3. Antioxidant Capacity Assays

#### 2.3.1. DPPH Assay

The DPPH radical scavenging activity was evaluated spectrophotometrically at 517 nm by measuring the decrease of DPPH absorbance after its reaction with the antioxidants [21,22]. DPPH solution was prepared in methanol at a concentration of 0.1 mM, whereas flavonoid solutions were prepared in a range of concentrations from 2 to 140 µmol/L. This range was selected for working in DPPH excess to deplete the H-donating capacity of flavonoids. For the assay, 100 µL of each standard solution was mixed with 100 µL of MeOH and 1.8 mL of the DPPH radical methanolic solution (0.1 mM). The mixtures were shaken and kept in the dark at room temperature. After 120 min, the DPPH absorbance was measured at 517 nm in triplicate. The results of the experiments were expressed as a percent of inhibition of DPPH radical, calculated as follows:

I% = [(A0 − A)/A0] × 100(1)

where A0 is the absorbance value in the absence of an antioxidant (blank), and A is the absorbance value in the presence of an antioxidant compound. Moreover, Trolox was used as a reference compound to build a calibration curve prepared using solutions at a concentration from 2 to 30 µmol/L to express the results as Trolox equivalents. Spectrophotometric analysis was carried out using a UV/Vis Spectrophotometer Cary 50 Scan (Varian Inc, Palo Alto, CA, USA).

#### 2.3.2. ABTS Assay

The ABTS+ scavenging activity test was performed for each flavonoid using the same standard solutions as in the DPPH assay. The radical cation ABTS+ was generated through the reaction of an aqueous solution of ABTS and potassium persulfate K_2_S_2_O_8_. In detail, 440 µL of a 140 mM K_2_S_2_O_8_ solution were added to 25 mL of a 7 mM ABTS solution and the mixture was left to react in the dark for 16 h at room temperature. The radical cation solution was diluted until an absorbance value of 0.70 ± 0.05 at 734 nm was reached [23]. For the test, 20 µL of each investigated flavonoid solution was added to 2 mL of ABTS+ solution, and the absorbance was measured after 6 min. The ABTS radical cation scavenging activity was determined by the following equation:

ABTS radical scavenging activity (%) = [(A0 − A)/A0] × 100(2)

where A0 is the absorbance value in the absence of an antioxidant (blank), and A is the absorbance value in the presence of a flavonoid. Also, for this assay, Trolox was employed as a reference compound to obtain the calibration curve and the analyses were performed using a UV/Vis Spectrophotometer Cary 50 Scan (Varian Inc, Palo Alto, CA, USA).

#### 2.3.3. FRAP Assay

The FRAP reagent was prepared according to Benzie and Strain method with slight modifications [24]: 2.5 mL of a 10 mM TPTZ (2,4,6-tripyridyl-s-triazine) acidic solution were mixed with 25 mL of acetic acid buffer (300 mM, pH 3.6) and 2.5 mL of 20 mM FeCl3 solution. The assay was performed by combining 30 µL of each standard flavonoid solution with 30 µL of water and 900 µL of FRAP reagent. The absorbance was measured at 534 nm after 15 min of reaction. The ferric reducing ability was determined as:

(%) = [(A0 − A)/A0] × 100(3)

where A0 is the absorbance value in the absence of an antioxidant (blank), and A is the absorbance value after the reaction. The results were also compared with those obtained from Trolox employment. UV determinations were carried out using a UV/Vis Spectrophotometer Cary 50 Scan (Varian Inc, Palo Alto, CA, USA).

### 2.4. Computational Details

The parameters for the peripolin (**1**), neoeriocitrin (**6**), melitidin (**3**) and naringin (**4**) were obtained calculated from HF/6-31 G* optimizations using the Gaussian09 D.01 package [25]. The General Amber Force Field (GAFF) and the Restrained Electrostatic Potential method were adopted to extrapolate non-bonding parameters and charges, respectively [26,27]. The here-adopted parametrization procedure represents a well-assessed protocol that was successfully adopted in previous studies [28,29,30].

Each species was inserted in a cubic box filled with TIP3P water molecules and later minimized. A gradually heating of the solute–solvent systems, from 0 K to 298 K, was performed for 5 ns in the NVT ensemble. The production phase consisted of 0.5 μs for all species and was conducted in NPT ensemble. All Molecular dynamics (MD) simulations were carried out using the AMBER16 software package [31], selecting a cutoff radii of 12.0 Å and the PME and SHAKE algorithms to use 0.002 fs of the integration step.

Geometry optimizations in the mean of density functional theory (DFT) were performed on open and closed conformations of each species. Such conformations were obtained from hierarchical clustering analysis of the MDs trajectories. The atoms of the antioxidant molecules were treated at B3LYP-D3 level of theory [32,33,34] and the 6-31 + G(d) basis set, as implemented G09 D.01 [25]. The nature of minima was verified by frequencies calculation on optimized structure (no negative frequency). Based on PCM scheme, [35], the implicit solvation method was employed during the geometry optimizations, selecting methanol and water as solvents. The protocol was extensively adopted in previous works on the antioxidant activity of natural products. [29,36,37,38,39]

The comparative antioxidant activity of investigated species was evaluated by considering the reaction of each molecule and the DPPH. In particular, attention was focused on the hydroxyl group at the 3′ positions for **1**, **3**, **4** and **6**, and at the 4′ positions, for **1** and **4**, according to the following reaction:

A-OH + DPPH → A-O∙+ DPPH-H(4)

## 3. Results

### 3.1. Structural Characterization of Peripolin

**Figure 2:**
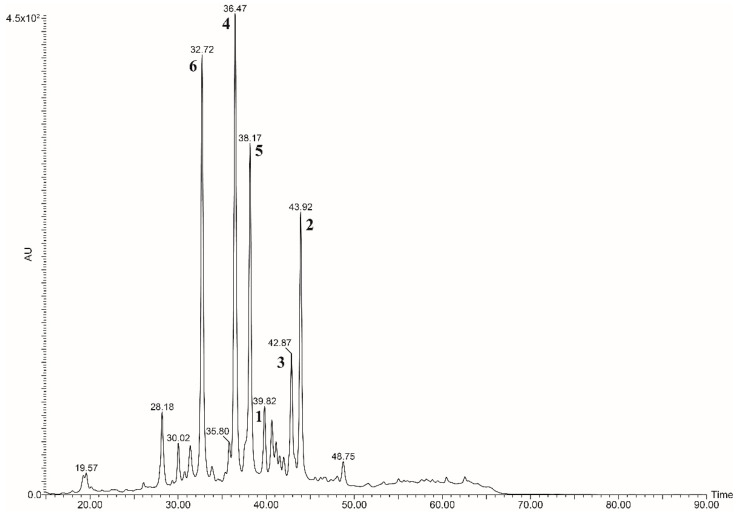
HPLC-UV flavonoid profile of bergamot.

The HPLC-UV profile of the flavonoid fraction of the bergamot fruit extract, obtained as reported in the sample preparation section, is shown in Figure 2. The most abundant peaks are related to classic di-glycosyl flavanones, such as neoeriocitrin (**6**, RT 32.72 min), naringin (**4**, RT 36.47 min) and neohesperidin (**5**, RT 38.17 min) [40].

The systematic presence of the neohesperidoside moiety (2-O-rhamnosyl glucoside) attached to position 7 of the flavanone *A*-ring is characteristic of the bergamot phenolic fraction. Furthermore, another peculiarity of the fruit is the unique presence of neohesperidoside flavanones esterified with the 3-hydroxy-3-methyl glutaric acid (HMG flavonoids). In 2009, the presence of such statin-like compounds [12] was reported for the first time in this fruit; in particular, the structures of the 6-O-glucosyl HMG derivatives of naringin and neohesperidin were elucidated through NMR and HRMS experiments: the signals of the latter compounds (i.e., melitidin (**3**) and brutieridin (**2**)) appear in Figure 1 at RT 42.87 min and 43.92 min, respectively. The chromatogram shows a further peak at RT 39.82, whose collected fraction negative mass spectrum discloses a prominent molecular ion at *m/z* value of 739. To achieve insights into the nature of this compound, fractionation of the entire flavonoid pool has been performed by preparative HPLC to attain the pure compound. The purification consisted of two semipreparative isocratic runs: the first run served as a gross purification step, which allowed for the collection of fractions 12 s wide, corresponding to 4.2 mL each (Figure S1). Those fractions were examined by analytical HPLC-UV (Figure S2) and mass spectrometry, then submitted to the further purification step, which was conducted narrowing the collection window at 5 s (Figure S3), to obtain the highest purity grade.

Once obtained, the pure compounds were in appropriate amounts (ca 100 mg, Figure S4), and further experiments were performed to elucidate the correct chemical structures. In particular, the compound at RT 39.82, which we previously named peripolin [41], was submitted to high-resolution MS and NMR experiments. The positive and negative HR-ESI MS spectra acquired at 60,000 resolution with an accuracy error within 2 ppm showed the protonated and the deprotonated species at *m/z* 741.2240 and *m/z* 739.2091, respectively (Figure S5), which matched the elemental composition of C_33_H_41_O_18_ with a delta error of 0.51 ppm for the protonated molecule [M + H]^+^ and C_33_H_39_O_19_ with 1.54 ppm delta error for the deprotonated molecule [M − H]^−^. 

Tandem mass spectrometry experiments provided insights into the structure of compound 1: the HR-ESI (+)MS/MS (Figure S6) obtained by the gas phase fragmentation of *m*/*z* 741.2240 showed some diagnostic ions, which provided some indication on the linkage of the ester moiety (Scheme S1). In particular, the base peak at *m*/*z* 289.0707 (C_15_H_13_O_6_, −0.03 ppm error) represents the protonated aglycon (eriodictyol). In contrast, the peak at *m*/*z* 453.1603 (B_2_^+^, C_18_H_29_O_13_, 0.02 ppm error) [42,43], generated by the loss of the neutral aglycon, suggests that the HMG ester is located on the glycosyl moiety. Further ions are those at 307.1022 (C_12_H_19_O_9_, −0.36 ppm error), which is a consecutive fragmentation of the B_2_^+^ ion produced by the loss of the rhamnose unit and the ion at *m*/*z* 595.1651 (C_27_H_31_O_15_, −1.12 ppm error), which comes from the loss of rhamnose (146 uma) from the precursor; these two ions indicates the HMG may be linked directly to the glucose. The gas-phase chemistry (Scheme S2) of the deprotonated molecular ion [M-H]^−^ at *m*/*z* 739.2091 shows the typical fragmentation of the HMG ester [12], i.e., the formation of [M-CO_2_-H_2_O-H (C_32_H_37_O_16_, 0.92 ppm error)]^−^ at *m*/*z* 677.2082, [M-C_4_H_6_O_3_-H (C_29_H_33_O_16_, −1.97 ppm error)]^−^ at *m*/*z* 637.1776 and [M-HMG-H (C_27_H_31_O_156_, 1.51 ppm error)]^−^ at *m*/*z* 595.1669, and, in addition, the ions at *m*/*z* 287.0553 (C_15_H_11_O_6_, 0.99 ppm error), representing the deprotonated aglycon eriodictyol, and its fragments at *m*/*z* 151.0028 (C_7_H_3_O_4_, 1.42 ppm error) and *m*/*z* 135.0442 (C_8_H_7_O_2_, 1.07 ppm error), which typically originate from the ^1,3^A_0_ and ^1,3^B_0_ fragments of the aglycone. Another ion typical of neoeriocitrin flavonoid is the species at *m*/*z* 501.1248 (C_21_H_25_O_14_, 1.88 ppm error), which originates from *m*/*z* 637.1780 fragmenting at the aglycon site. The above fragmentations indicate that the HMG moiety might be linked at either C-3, C-4 or C-6 position of the glucose unit. To confirm this hypothesis, high-resolution NMR experiments were performed on pure compounds. The ^1^H-NMR and ^13^C-NMR experiments (Table 1, Figures S7–S10) provide structural information about the molecule, including the position of the linkage of the 3-hydroxy-3-methylglutaryl moiety on the sugar.

It may be seen from the ^1^H-NMR spectrum that the signal of the methylene protons at the primary position of glucose, identified as a doublet of doublets at δH 4.19 (1H, dd, J = 2.0, 11.9 Hz, H-6″a) and δH 4.19 1H, dd, J = 7.5, 11.9 Hz, H-6″b), are shifted at lower resonance fields respect to that of the equivalent protons of the neoeriocitrin or any other 7-O-neohesperidoside flavanone with primary alcoholic position free: The latter, in fact, are usually found in the range of 3.3–3.9 ppm. The shift is due to the carbonyl of the ester moiety. Other signals suggesting the presence of the HMG moiety were present at δH 1.27 (3H, s, H-6⁗) and δH 2.52–2.72 (4H, m, H-2⁗ and H-4⁗): they represent, respectively, the methane and methylene protons of the glutaryl side. The acetal moiety of the glucose unit of the neohesperidoside is represented typically by the signal found at δH 5.08 (1H, d, J = 7.6 Hz, H-1″). The ^13^C spectrum displayed a quadruplet, two triplet and three singlet signals assigned, respectively, to C-6⁗ (δC, 27,4), C-2⁗, C-4⁗ (δC 45.0, 46.5) and C-1⁗, C-3⁗, C-5⁗ (δC 172.5, 70.8, 175.7)

Furthermore, the ^1^H-NMR data (Table 1) justifies the structure of eriodictyol aglycone: the multiplets (δH 6.176, 1H, m, H-6 and δH 6.14, 1H, m, H-8) are easily assigned to the aglycone protons at position 6 and 8 respectively (A ring). The other aromatic protons of the B ring are shown in the spectrum as multiplets (δH 6.79, 1H, m, H-2′; δH 6.80, 1H, m, H-5′; and δH 6.93, 1H, m, H-6′). The HMQC spectrum (Figure S10) correlates the aromatic protons to the ^13^C signals at δC 116.65 (d, C-2′), 112.4 (d, C-5′), 114.9 (d, C-6′), 97.0 (d, C-6) and 98.1 (d, C-8). All other structural characteristics are summarized in Table 1 and are clearly inferred by NMR correlation experiments between protons (HHCOSY) and ^13^C (HMQC), which are displayed in Figures S9 and S10.

The presence of the 3-hydroxy-3-methyl glutaryl ester moiety was also confirmed by basic hydrolysis experiments, which were carried out using NaOH 0.1 M; the experiments showed the formation after only 30 min of neoeriocitrin. It is worth noting that similar experiments conducted with Na_2_CO_3_ and NaHCO_3_ did not yield the expected basic hydrolysis product, as previously reported [12], instead tending toward oxidative degradation due to the presence of the catechol moiety.

Another set of experiments was carried to elucidate the sequence of sugars in the molecule: the isolated compound **1**, was treated with the enzyme neohesperidase; after 4 h the enzymes eliminated the rhamnose unit producing eriodictyol 7-O-(6″-(3‴-hydroxy-3‴-methylglutaryl)-β-glucoside) that has been fully characterized by HRMS experiments (data not shown). On the other hand, after 20 h of reaction time, the only product present in the reaction was eriodictyol (Figure S11).

### 3.2. Antioxidant Activity of HMG Flavonoids

**Figure 3:**
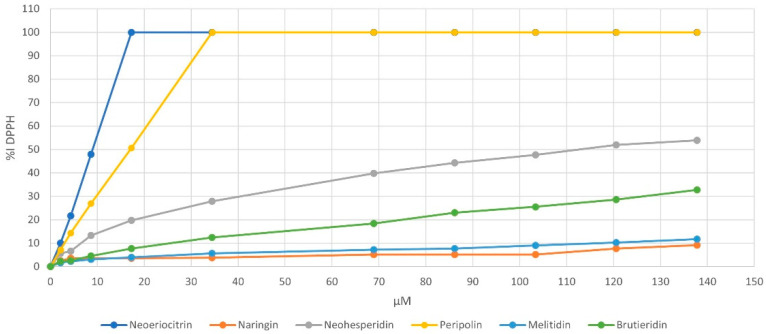
Inhibition of DPPH (%) versus flavonoids concentration.

Once the structural characterization of peripolin was completed, a series of experiments were carried out to explore the antioxidant characteristics of the flavonoids in bergamot. The antioxidant ability of pure neoeriocitrin, naringin, neohesperidin, melitidin, brutieridin, and the new compound peripolin, was determined and compared by applying the three most common assays: DPPH, ABTS and FRAP. The first two tests are based on an antioxidant molecule’s reducing ability towards a radical species. During the reaction between the DPPH radical and the ABTS radical cation, the antioxidant molecule quenched the radicals by transferring one or more hydrogen atoms; this led to the oxidation of the reacting molecule and the reduction of the radicals, with a resulting decrease in the solution absorbance. For all investigated flavonoids, the results obtained from the two assays are very similar and highlighted a strong concentration-dependent antioxidant activity and a radical scavenging ability related to their chemical structure. As expected, for both assays, neoeriocitrin and peripolin showed the highest activity against free radicals. Figure 3 shows the change in % DPPH reduction depending on the concentration of all investigated flavonoids. The order of radical scavenging activity is neoeriocitrin ≥ peripolin ˃ neohesperidin ˃ brutieridin ˃ melitidin ˃ naringin.

**Figure 4:**
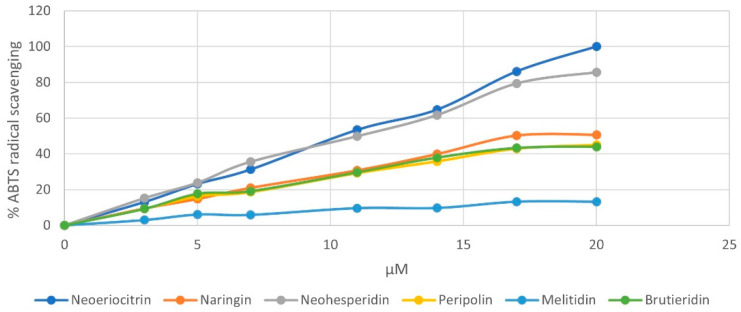
Reactivity behavior of pure flavonoids from bergamot against ABTS radical ion.

This trend confirms how the free radical scavenging ability exhibited by flavonoids depends on the type of substituents on the A, B and C rings, on their number and position. As with neoeriocitrin, which exerts the highest antioxidant activity, the catechol structure present at the B ring provides greater stability of phenoxyl radicals due to increased electron delocalization, resulting in high antioxidant power. It should be noted that peripolin, carrying the HMG portion, achieve the same activity as neoeriocitrin. All other flavonoids possess a considerably lower antioxidant capacity. Neohesperidin, with a hydroxy group at C-3′ and a methoxy group at C-4′ showed higher antioxidant activity than brutieridin, which possesses the same aglycone structure but the 3-hydroxy-3-methylglutaric acid moiety. Furthermore, naringin, which has just a hydroxy group at C-3′ exhibits the lowest antioxidant capacity together with melitidin. Similar behavior is observed with respect to the ABTS radical cation, but in this case, neoeriocitrin, naringin and neohesperidin all appear to exert greater activity than their HMG conjugates, even if among the latter, peripolin keeps on having the best antioxidant activity (Figure 4).

For both assays, the results were also expressed in terms of IC_50_ (µmol/L), which is the concentration of the antioxidants necessary to decrease the initial free radical concentration by 50% (Table S1).

**Figure 5:**
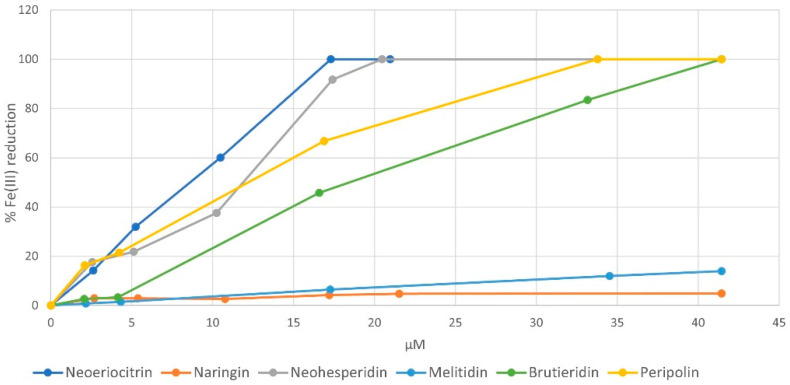
Ferric-reducing ability of pure flavonoids.

It is interesting to note that neoeriocitrin and peripolin not only have the lowest IC_50_ values, but they are very similar in both tests, suggesting that their antioxidant action is independent of the nature of the free radical. Finally, comparing the activity of these flavonoids with that of Trolox, selected as the reference compound, it can be noted that at the same antioxidant concentration, neoeriocitrin and peripolin exhibited a stronger radical scavenging capacity (Table S1). With regard to the FRAP assay, which measures the change in absorbance at 593 nm resulting from the formation of a blue-coloured Fe (II)-tripyridyltriazine compounds from the colourless form of Fe (III) reduced by the action of electron-donating antioxidants, the value of IC_50_ confirmed the trend already discussed above for radicals DPPH and ABTS scavenging (Table S1). In addition, the new HMG flavonoid, peripolin, again displays high antioxidant properties, revealing its action already at low concentrations. The ferric-reducing ability of the investigated flavonoids is presented in Figure 5: here, it is possible to observe that neoeriocitrin and peripolin, together with neohesperidin, showed the highest activity.

### 3.3. Computational Studies of the Antioxidant Capacity of HMG Flavonoids

To further support the experimental results, a set of computational studies focused on compounds **1**, **3**, **4** and **6**. Such species were selected based on the measured radical scavenging activity in the presence of DPPH (Figure 3) and represent the extremities of the series (see Section 3.2). In addition, this choice allowed us to evaluate the influence of the HMG group on the antioxidant activity present in species **1** and **3** and relatively missing in **6** and **4**, respectively. Molecular dynamics simulations were performed in the presence of explicit water molecules with the aim to evidence the assumed possible conformations during the simulation time of 0.5 μs. In fact, the rotatable bonds present in the chemical skeleton of the examined flavonoids (see Scheme S3) can promote the switching between the open-closed conformations, which in turn can be helpful to obtain further insights into their antioxidant behavior.

In particular, a careful analysis covered the dihedral angles ω1 and ω2 (Scheme S3), common to all the examined species, and the ω3, only related to **1** and **3** having the HMG moiety in the 3-hydroxy-3-methyl-glutaryl-neohesperidoside sugar region). The frequency distribution of the values of dihedral angles from MD trajectories for all the examined molecules is reported in Figure S12.

**Figure 6:**
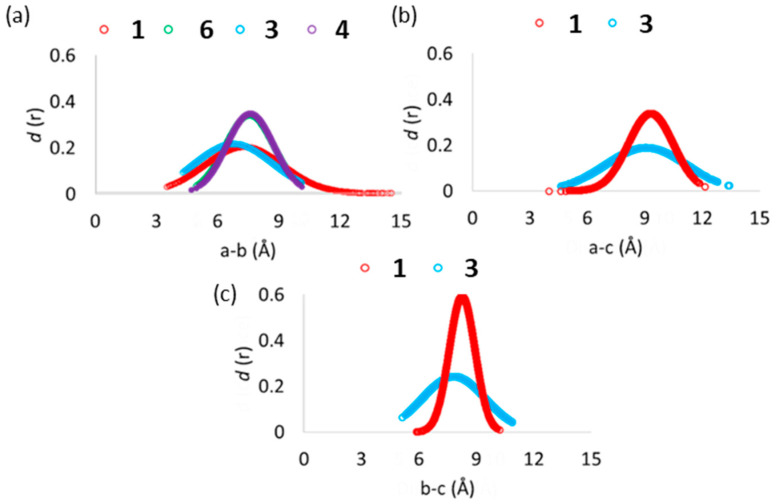
Distance distributions obtained from the MD trajectories for the groups comprising compounds **1**, **3**, **4** and **6**. Labels (**a**–**c**) were assigned according to Scheme S3.

The ω1, corresponding to the spatial arrangement of the sugar ring (labelled with “**b**”, see Scheme S3) with respect to the substituted one, is located in a range from −120° to −60° in **1** and thus exhibits a different behavior from the **3** and **4** species, for which the same dihedral assumes very similar values (Figure S12). The ω2 outlines the spatial disposition of the O-neohesperidoside fragment in relation to the A and C rings of the flavone moiety. Its value does not significantly contribute to the antioxidant molecules’ conformational diversity since an equal frequency distribution within the 360 degrees was observed. (see Figure S12) A closer look at the conformation behavior of ω3 in **1** and **3**, reveals that the HMG moiety frequently lies in a range from −80.0° to −40.0° respective to the O-neohesperidoside ring. This result suggests that **1** and **3** can assume a “closed” conformation to favor intramolecular hydrogen-bond interactions between the **b** ring and the carboxylic acid group of HMG (see Figure 6). 

Analysis of the ring–ring distances in Figure 6 further confirms such behavior. Indeed, the examination of the relative distance of the centers of mass of the three different rings revealed a more frequent facing of HMG group to the **b** ring than to the A and C rings of the flavone moiety (labelled with **a** in Scheme S3), in the case of **1** (Figure 6c). Furthermore, it is interesting to note that despite the presence of HMG, the conformational behavior of melitidin rings substantially differs from that of peripolin and could contribute to explaining the difference in measured antioxidant activity (Figure 3). However, in general, ring–ring interactions are present in the MD simulations also for the other investigated systems, thus suggesting that in solutions, the antioxidant molecules can display closed or folded and open or unfolded conformations.

The observations arising from MD analysis led to considering both folded and unfolded conformations for the subsequent DFT-based investigations. In particular, starting from the most populated clustered geometry, each species’ open and closed conformations were optimized in water and methanol (see the optimized geometries in Figure S13).

The visual inspection of the optimized geometries revealed the presence of a higher number of hydrogen bonds in the closed conformations of each species, irrespective of the nature of the solvent. This can be the origin of the calculated higher energetic stability of closed conformations energies (see Figure S13).

To estimate and compare the antioxidant properties, the frontier orbitals and the reaction energy with DPPH (see Section 2.4 for details) were calculated (see Figure S14). All the species have the highest occupied molecular orbital (HOMO) delocalized on the π system of the A and C rings. In contrast, the lowest unoccupied molecular orbital (LUMO) results on the B ring (see Figure S13). In the case of the **1** and **6** molecules, a smaller HOMO-LUMO gap was observed, mainly caused by higher HOMO energy, which represents a good electronic parameter for explaining the propensity of a molecule to act as an electron donor. Therefore, the low values of HOMO energy can be related to a weak electron donating ability. In contrast, higher HOMO energy can be linked to a molecule with good electron-donor behavior. In the case of **1** and **6**, this is caused by the presence of the additional hydroxyl group at the 3′ positions. This HOMO-LUMO gap is not affected by the conformation of the antioxidant.

## 4. Discussion

Bergamot is already known as the richest citrus fruit for concerns the phenolic content. It makes its extract suitable to be the main ingredient of pills and food supplements, particularly for lowering or maintaining blood cholesterol levels. As shown above, this characteristic is given mainly by the complexity of the phenolic molecular profile; in addition, these molecules are also present in considerable amounts, and we demonstrated that the peripolin (**1**), together with the other flavonoids, may behave potentially as strong antioxidants. Hence, we performed a quantitative assay of **1** and the other flavonoids in commercial bergamot beverages and bergamot extracts in order to estimate their amounts. The assay was performed by means of ESI-MS/MS, using a triple stage quadrupole in multiple reactions monitoring scan mode as an analyzer and caffeic acid as an internal standard. Table 2 shows the amount found for each molecule in the different matrixes (see experimental for the detailed procedure).

The analysis performed on commercial juices and nutraceutical ingredients revealed that considerable amounts of flavonoids might also be found in products subjected to industrial processes. In particular, the content of the main phenolic fraction in the beverage samples prepared with 100% bergamot juice reach concentrations near 0.2%, while in the powders used as an ingredient for nutraceuticals, the concentration may arrive at 50%. As a consequence, bergamot can be considered a fully-fledged nutraceutical food, giving a further boost to its use as standalone food or as a source of nutraceuticals in food supplements. In particular, peripolin, together with brutieridin and melitidin, which are uniquely found in bergamot fruit, may be used as quality markers for bergamot-based products.

It is worth noting that either neoeriocitrin or peripolin act as strong antioxidant species compared to Trolox, as highlighted in Table S1. This may reveal new perspectives for bergamot relative to the use of this food as a nutraceutical in comparison to other foods whose health benefits are regulated by laws [44].

Regarding the reactivity with DPPH, a thermodynamically favored ΔG_rea_ for **1** and **6** has been calculated (see Figure S14b). In particular, the lowest values were obtained for the reaction with the hydroxyl group at the 3′ and at 4′ positions, for **1** and **6**, respectively. Interestingly, these values concern the open conformation of the antioxidants. A possible explanation of such a result can be that, since in the closed conformation, the hydroxyl groups are involved in many intramolecular interactions, it is more energetically demanding to abstract the hydrogen from the antioxidant. For the **3** and **4** molecules, high values were obtained for both conformations, in agreement with the % inhibition of DPPH depicted in Figure 3. The relative values reported in Figure S14b, suggest a better activity of **6** with respect to **1**, unlike **3** and **4,** which in turn showed an unfavorable thermodynamic behavior and a lower % inhibition of DPPH. It is worth noting that the current calculations do not consider any kinetic control contribution to the reaction, which might play a crucial role in the DPPH assay.

## 5. Conclusions

This study reports the full structural characterization of Peripolin, a new 3-hydroxy-3-methylglutaryl flavonoid in bergamot fruit. The chemical structure was elucidated by nuclear magnetic resonance and high-resolution tandem mass spectrometry experiments, which revealed the presence of the glutaryl moiety linked to the primary position of a neohesperidoside unit attached to the position 7 of eriodictyol. The presence of catechol on the aglycone moiety suggested a strong antioxidant activity of the new molecule. The latter was demonstrated by different chemical assays, such as DPPH, ABTS and FRAP tests, carried out on pure bergamot flavonoids. In particular, the classic DPPH test highlighted the highest antioxidant activity for neoeriocitrin and peripolin. This evidence has been supported by computational studies, particularly by investigating the possible conformations adopted by the antioxidants in the presence of water molecules and by calculating ΔG_rea_ for the DPPH test. Furthermore, a quantitative assay of the main bergamot flavonoids, performed by UHPLC-ESI-MS/MS, demonstrated that peripolin is one of the active principles with the highest concentration in commercial bergamot juices and in bergamot extracts used in nutraceutical production.

## Figures and Tables

**Table 1:** ^1^H NMR and ^13^C NMR Spectroscopic Data for Compounds **1**.

Peripolin (1)		
Position	δ_H_ (*J* in Hz)	δ_C_ Mult
2	5.34 dd (2.90, 12.7)	80.6, CH
3a	3.12 dd (12.7, 17.2)	44.0, CH_2_
3b	2.74 dd (2.9, 17.2)	44.3, CH_2_
4		198.4, qC
5		164.9, qC
6	6.16 m, ar	97.0, CH
7		166.5, qC
8	6.14 m, ar	98.1, CH
9		164.6, qC
10		105.1. qC
1′		131.7, qC
2′	6.79 m, ar	116.5, CH
3′		147.0, qC
4′		146.6, qC
5′	6.80 m, ar	119.4, CH
6′	6.93 m, ar	114.9, CH
1″	5.08 d (7.6)	99.5, CH
2″	3.66 dd (7.7, 9.0)	78.9, CH
3″	3.60 dd (8.3, 9.0)	79.1, CH
4″	3.36 dd (8.3, 10.4)	71.8, CH
5″	3.69 ddd (2.0, 7.5, 11.9)	75.5, CH
6″a	4.40 dd (2.0, 11.9)	64.6, CH_2_
6″b	4.19 dd (7.5, 11.9)	
1‴	5.25 d (1.4)	102.5, CH
2‴	3.93 dd (1.4, 3.2)	72.2, CH
3‴	3.0 m	72.3, CH
4‴	3.40 dd (1.6, 9.5)	74.1, CH
5‴	3.90 ddd (3.9, 6.2, 9.5)	70.0, CH
6‴	1.30 d (6.2)	18.2, CH_3_
1⁗		172.5, qC
2⁗	2.72–2.52 m	45.0, CH_2_
3⁗		70.7, qC
4⁗	2.72–2.52 m	46.5, CH_2_
5⁗		174.9, qC
6⁗	1.27 s	27.7, CH_3_

**Table 2:** Concentrations of the main flavonoids in bergamot samples (PE powder extract, SB sample beverage, PB prototype beverage).

	1	2	3	4	5	6
PE1 ^‡^	6.0 ± 0.1 ^‡^	40 ± 1 ^‡^	28 ± 1 ^‡^	164 ± 3 ^‡^	160 ± 9 ^‡^	78 ± 4 ^‡^
PE2 ^‡^	5.0 ± 0.7 ^‡^	39 ± 4 ^‡^	32 ± 4 ^‡^	152 ± 4 ^‡^	129 ± 5 ^‡^	71 ± 2 ^‡^
PE3 ^‡^	4.2 ± 0.3 ^‡^	29.0 ± 0.2 ^‡^	15 ± 2 ^‡^	138 ±7 ^‡^	130 ± 3 ^‡^	85 ± 1 ^‡^
PE4 ^‡^	3.1 ± 0.2 ^‡^	28 ± 1 ^‡^	16 ± 1 ^‡^	136 ± 6 ^‡^	120 ± 8 ^‡^	85 ± 5 ^‡^
PE5 ^‡^	3.0 ± 0.2 ^‡^	28 ± 1 ^‡^	16 ± 1 ^‡^	145 ± 5 ^‡^	119 ± 9 ^‡^	90 ± 5 ^‡^
SB1 * (30% bergamot juice)	23.5± 0.1 *	53.1 ± 0.1	29.6± 0.2	76.7 ± 0.3 *	50.4 ± 0.2 *	57.8 ± 0.2 *
PB1 * (Stevia and 30% bergamot juice)	13.7 ± 0.6 *	68.4 ± 0.3 *	36.5 ± 0.2 *	106.1 ± 2 *	53.6 ± 0.2 *	79.1 ± 0.3 *
SB2 * (100% bergamot juice 2019)	56.1± 0.2 *	184 ± 2 *	66 ± 0.3 *	678 ± 13 *	611 ± 22 *	503 ± 7 *
SB3 * (100% bergamot juice 2020)	66.2 ± 0.1 *	187 ± 4 *	92 ± 0.1 *	354 ± 14 *	234± 2 *	334 ± 12 *
SB4 * (100% bergamot juice 2021)	108.1 ± 2 *	380 ± 12 *	162 ± 11 *	617 ± 13 *	332 ± 8 *	520 ± 17 *

^‡^ Values expressed as g per kg. * Values expressed in mg/kg.

## Data Availability

All data are contained within the article.

## Supplementary Materials

The following are available online at https://www.mdpi.com/article/10.3390/antiox11101847/s1.
