# Supplementary material for: Structural Characterization of Peripolin and Study of Antioxidant Activity of HMG Flavonoids from Bergamot Fruit
Source: Antioxidants (Basel). 2022 Sep 20;11(10):1847. doi: 10.3390/antiox11101847 (PMC9598738; doi:10.3390/antiox11101847)
Supplement: Supplementary file 1 [file antioxidants-11-01847-s001.zip › antioxidants-1900850-supplementary-1.pdf]

Structural characterization of Peripolin and Study of Antioxidants Activity of HMG-Flavonoids from Bergamot Fruit

Supplementary material

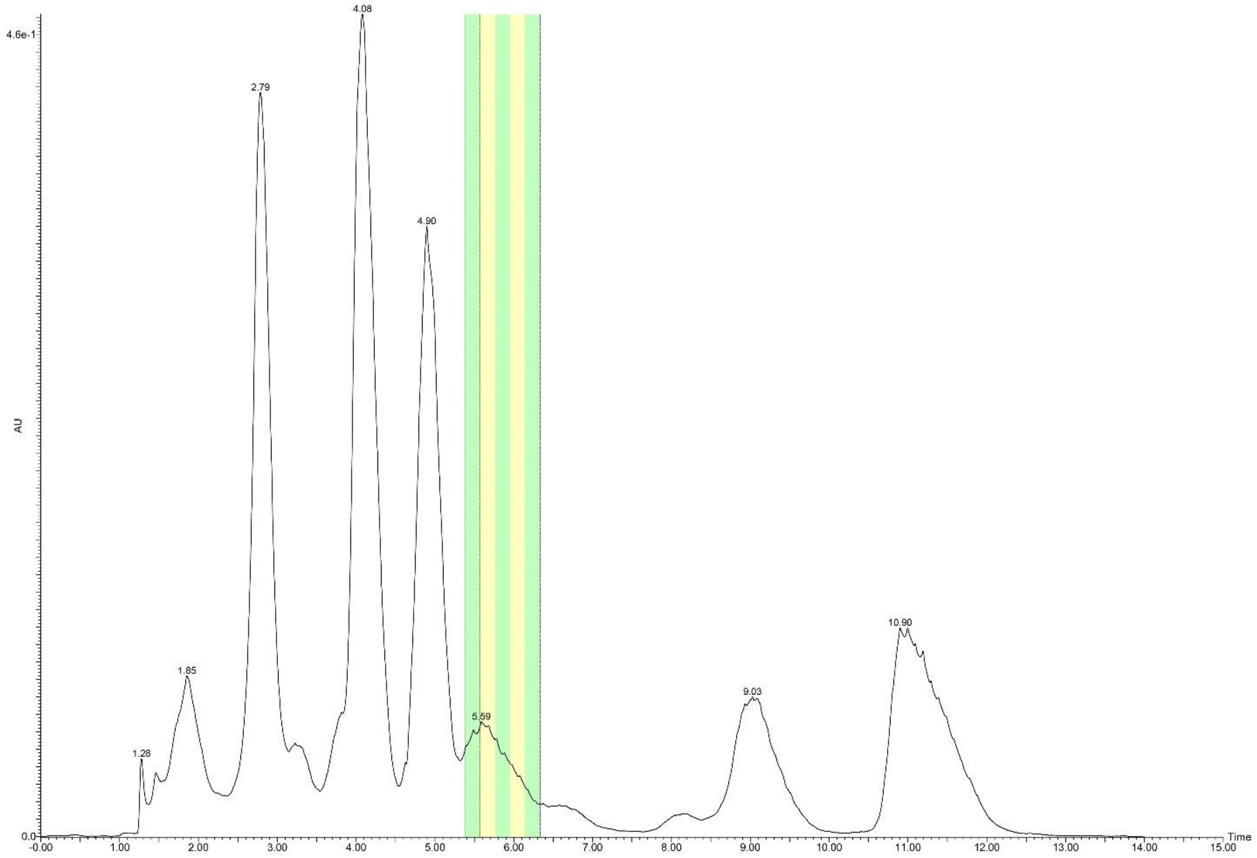

Figure S1. Semipreparative HPLC-UV chromatogram from gross fractionation of the bergamot flavonoid pool. Each fraction collected is represented by yellow and green column corresponding to a collected volume of 4.2 mL.

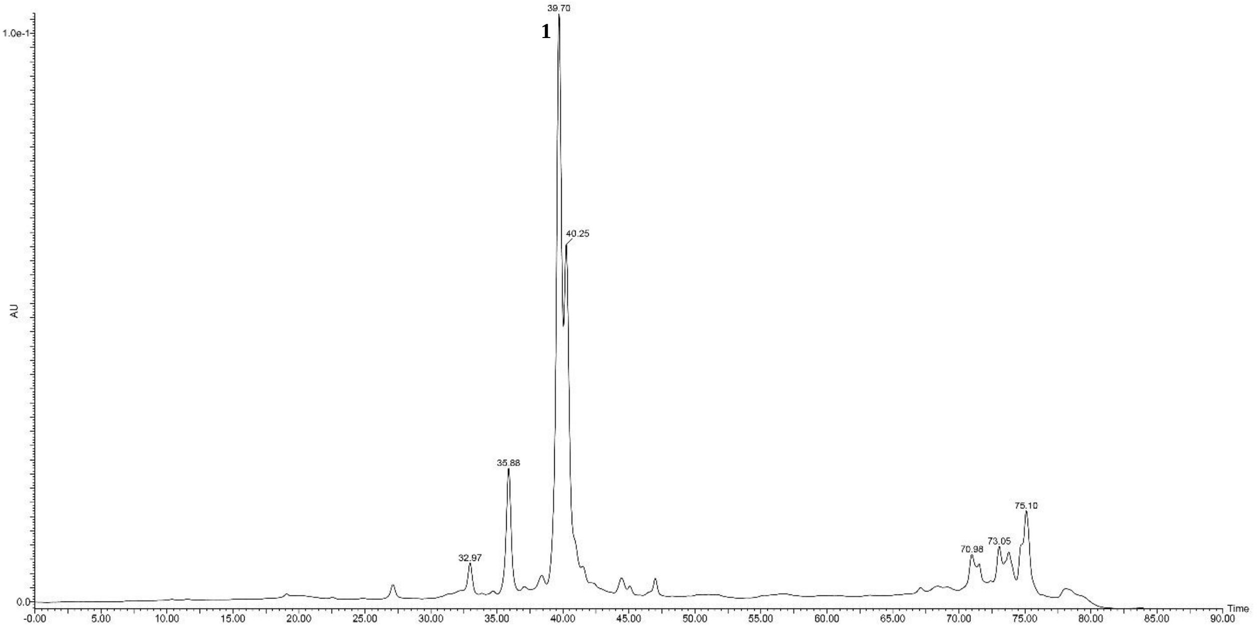

Figure S2. HPLC-UV chromatogram of the partially purified fraction containing peripolin (**1**)

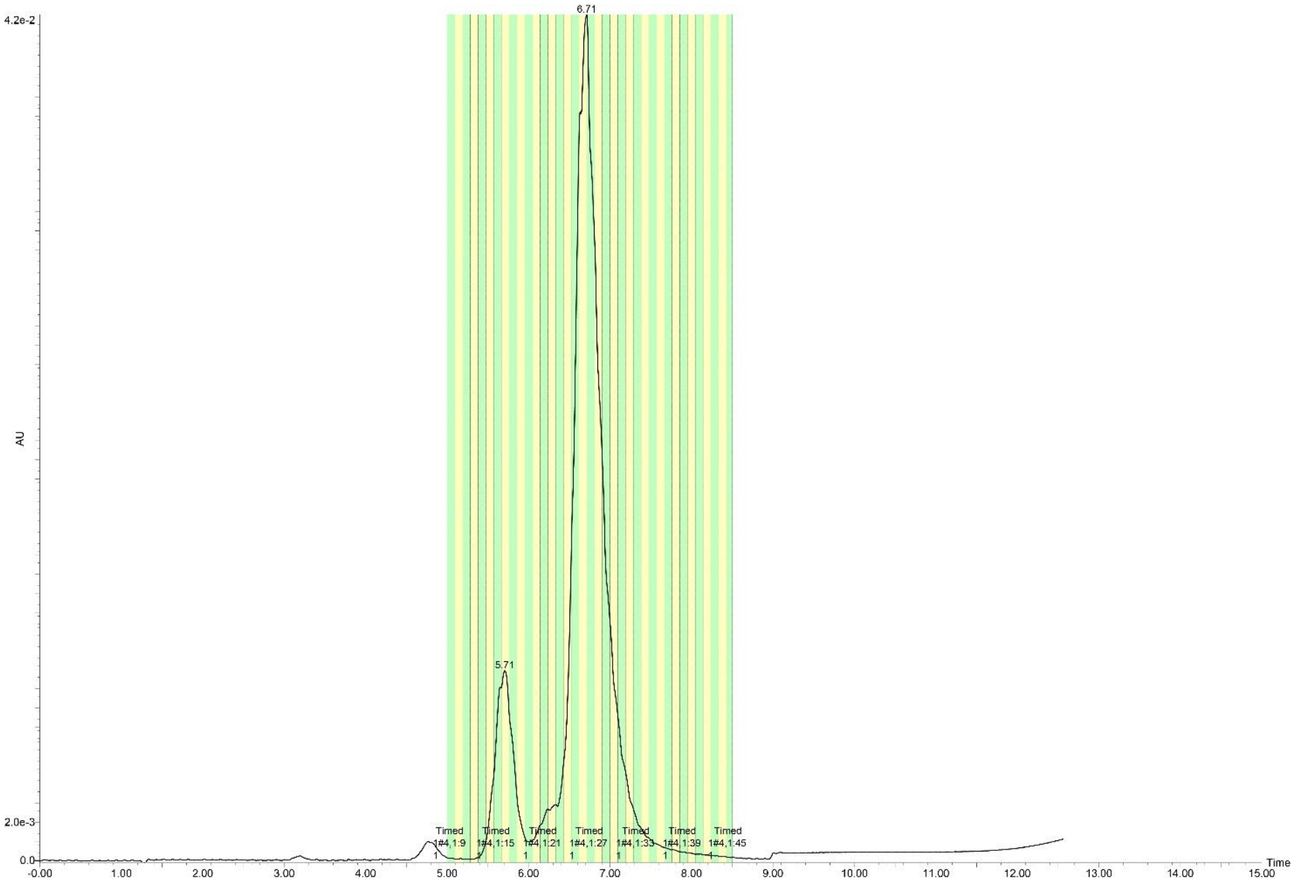

Figure S3. Semipreparative HPLC-UV chromatogram of the partially purified solution containing peripolin from the fine fractionation step. Each fraction collected is represented by yellow and green column corresponding to a volume of 2 mL.

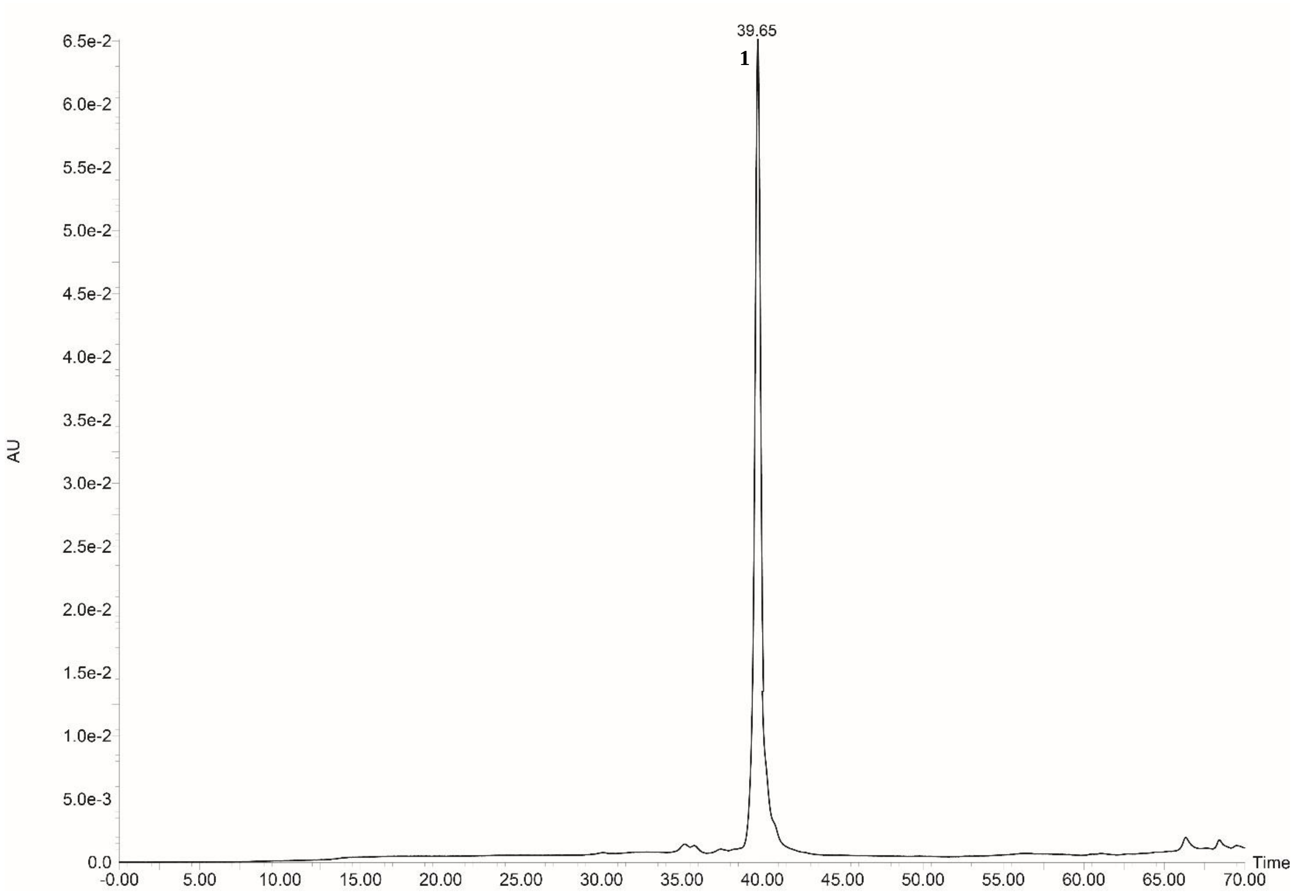

Figure S4. HPLC-UV chromatogram of the pure peripolin (**1**)

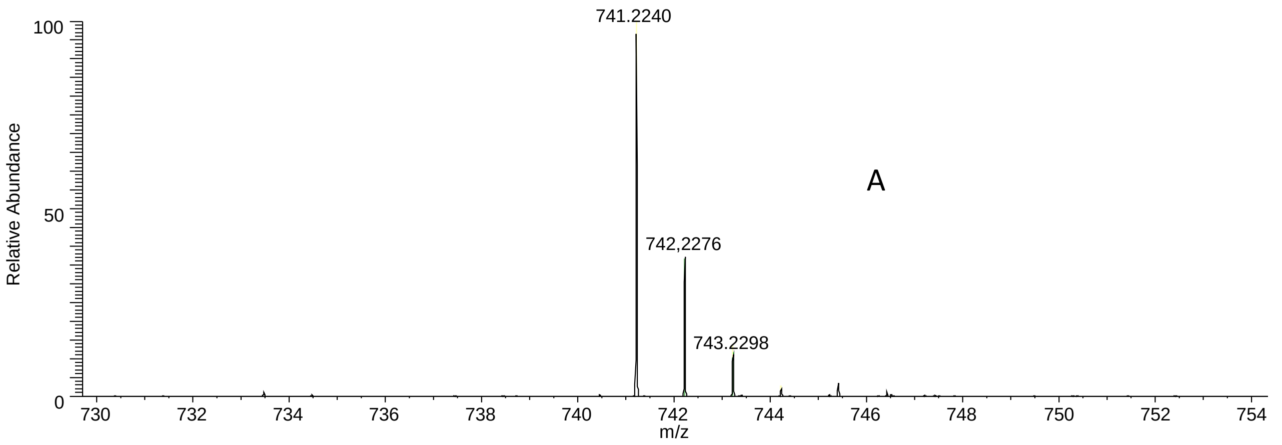

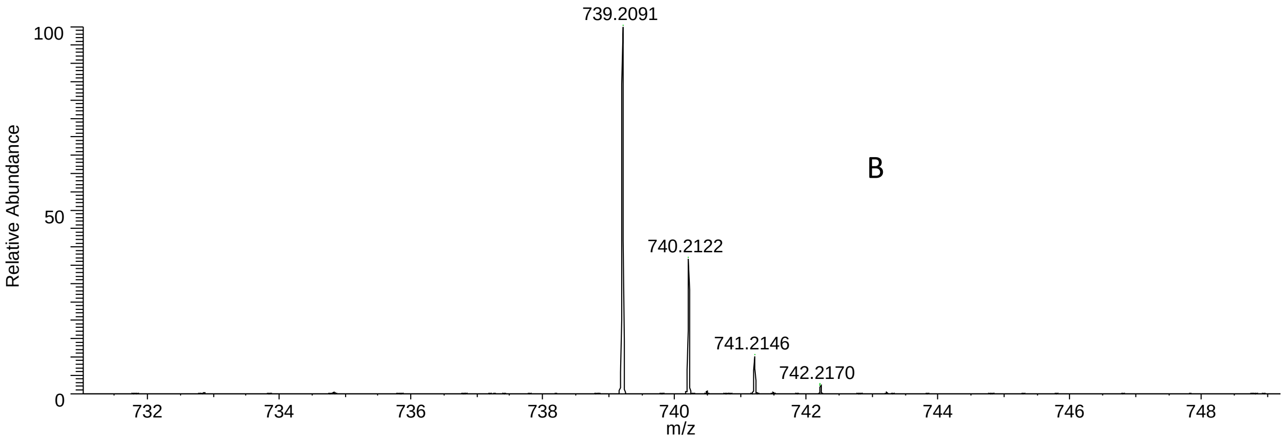

Figure S5. HR-ESI MS spectra of pure Peripolin (**1**) in positive (**A**) and negative (**B**) ion mode.

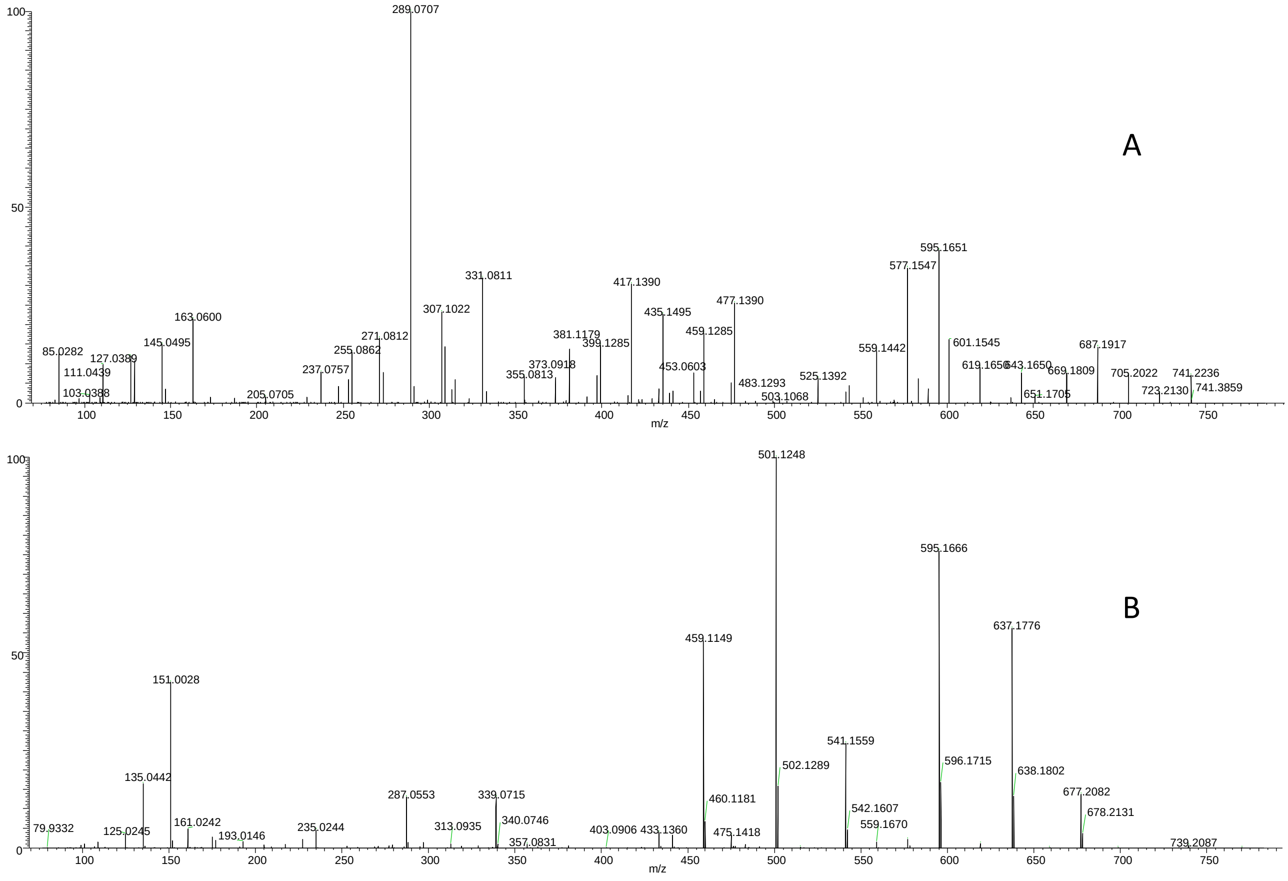

Figure S6. HR-ESI MSMS spectra of pure Peripolin (**1**) in positive (**A**) and negative (**B**) ion mode.

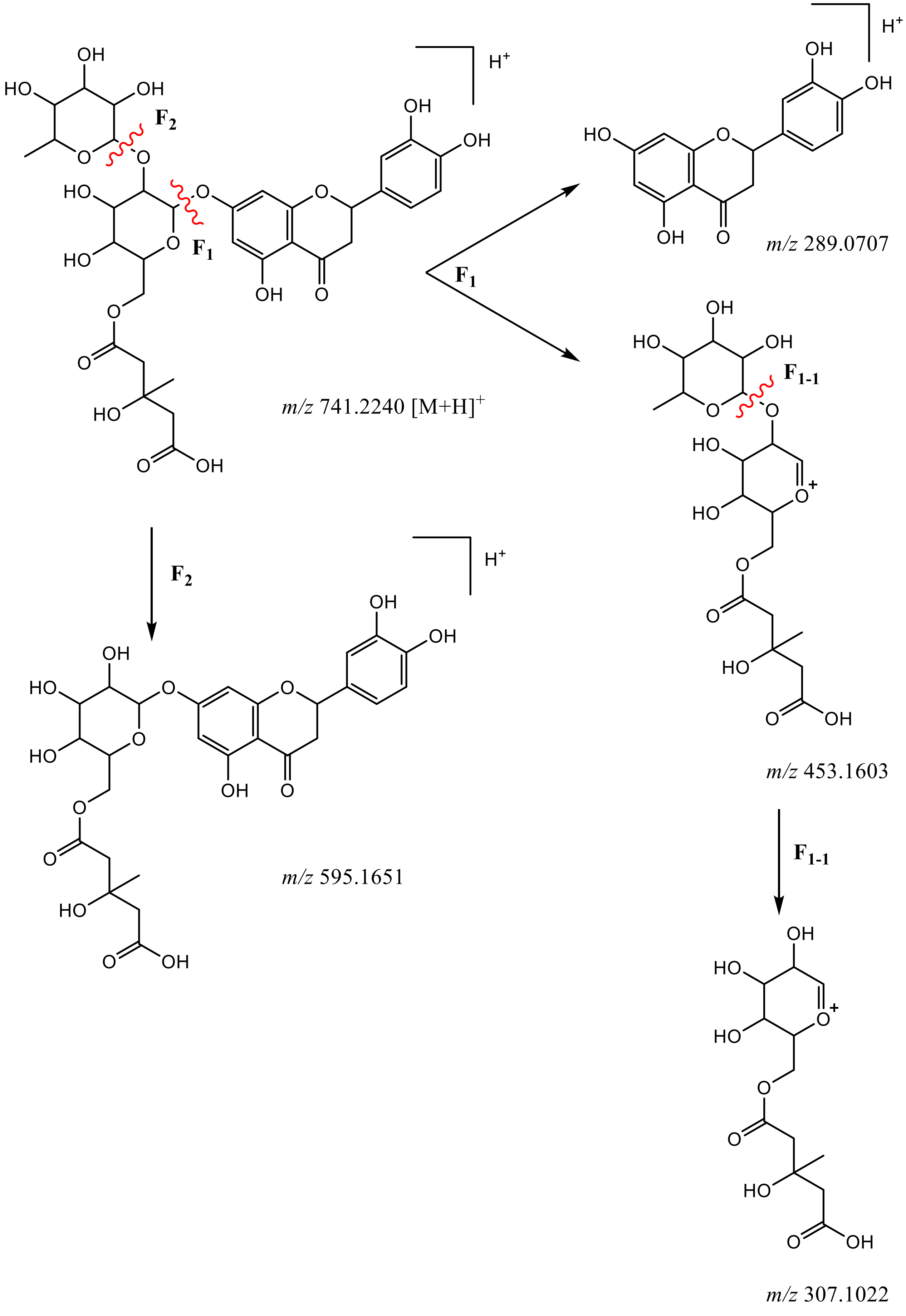

Scheme S1. Gas phase fragmentation of protonated peripolin in positive mode.

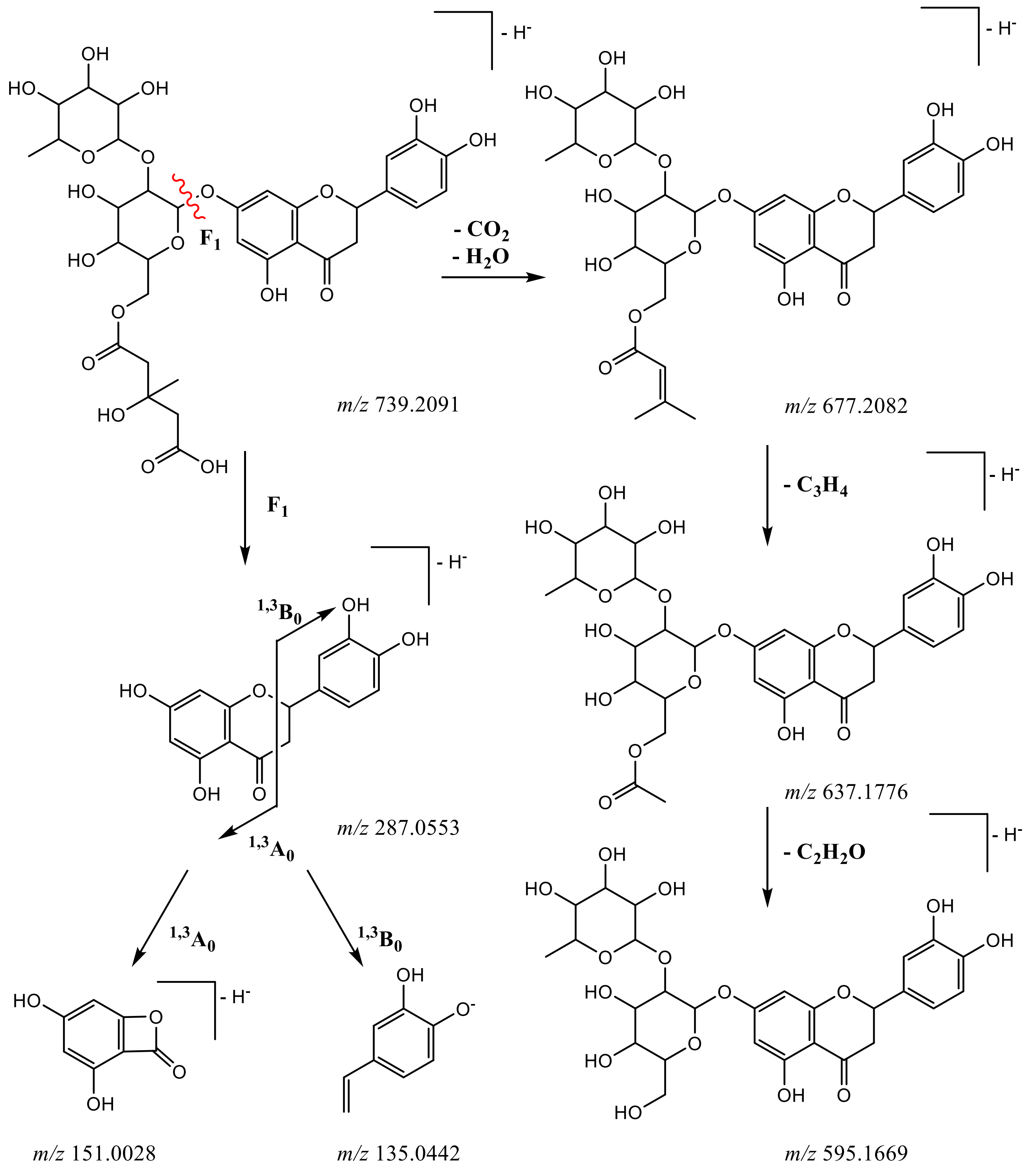

Scheme S2. Gas phase fragmentation of deprotonated peripolin in negative mode.

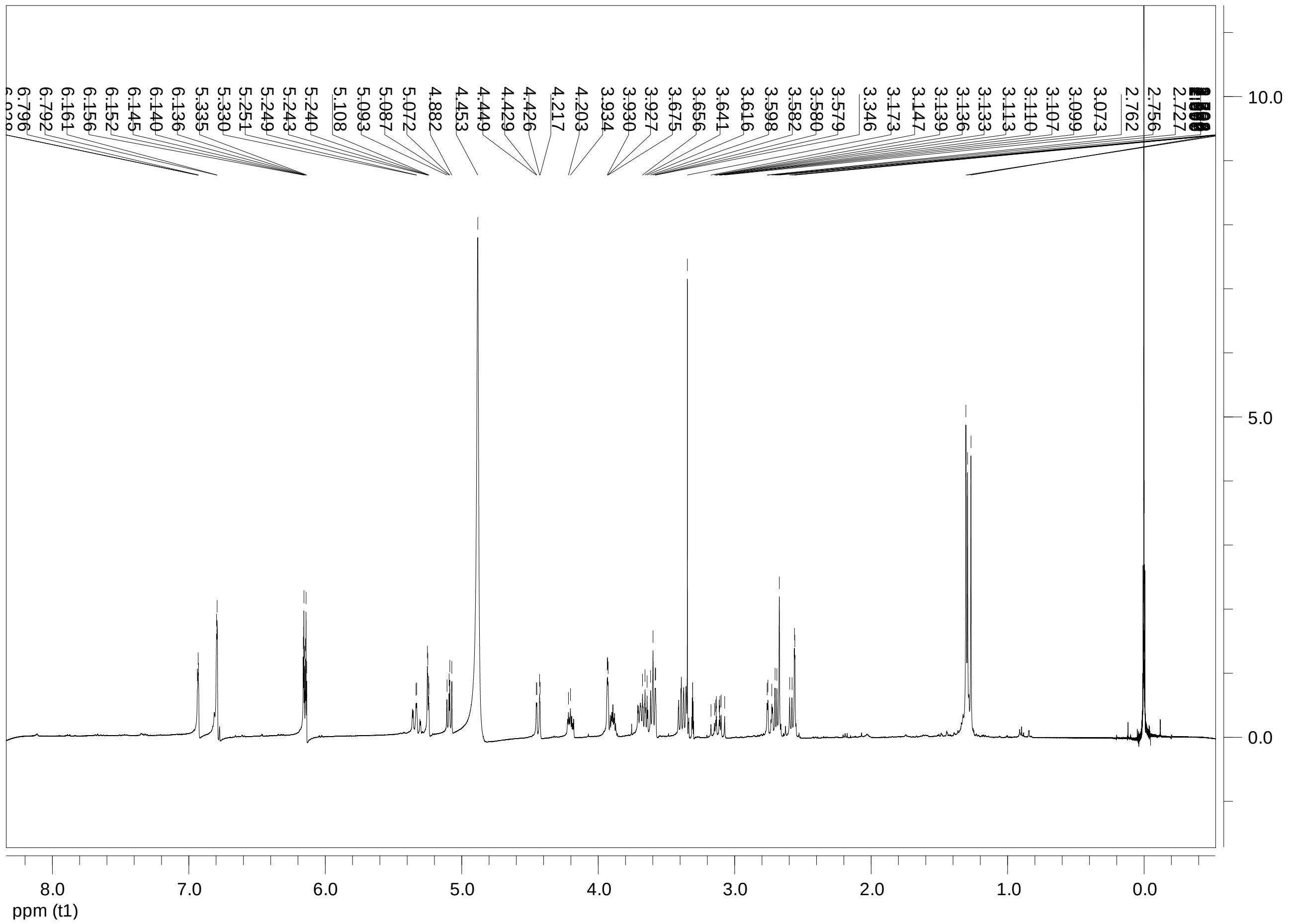

Figure S7. ^{1}H-NMR spectrum of pure Peripolin (**1**).

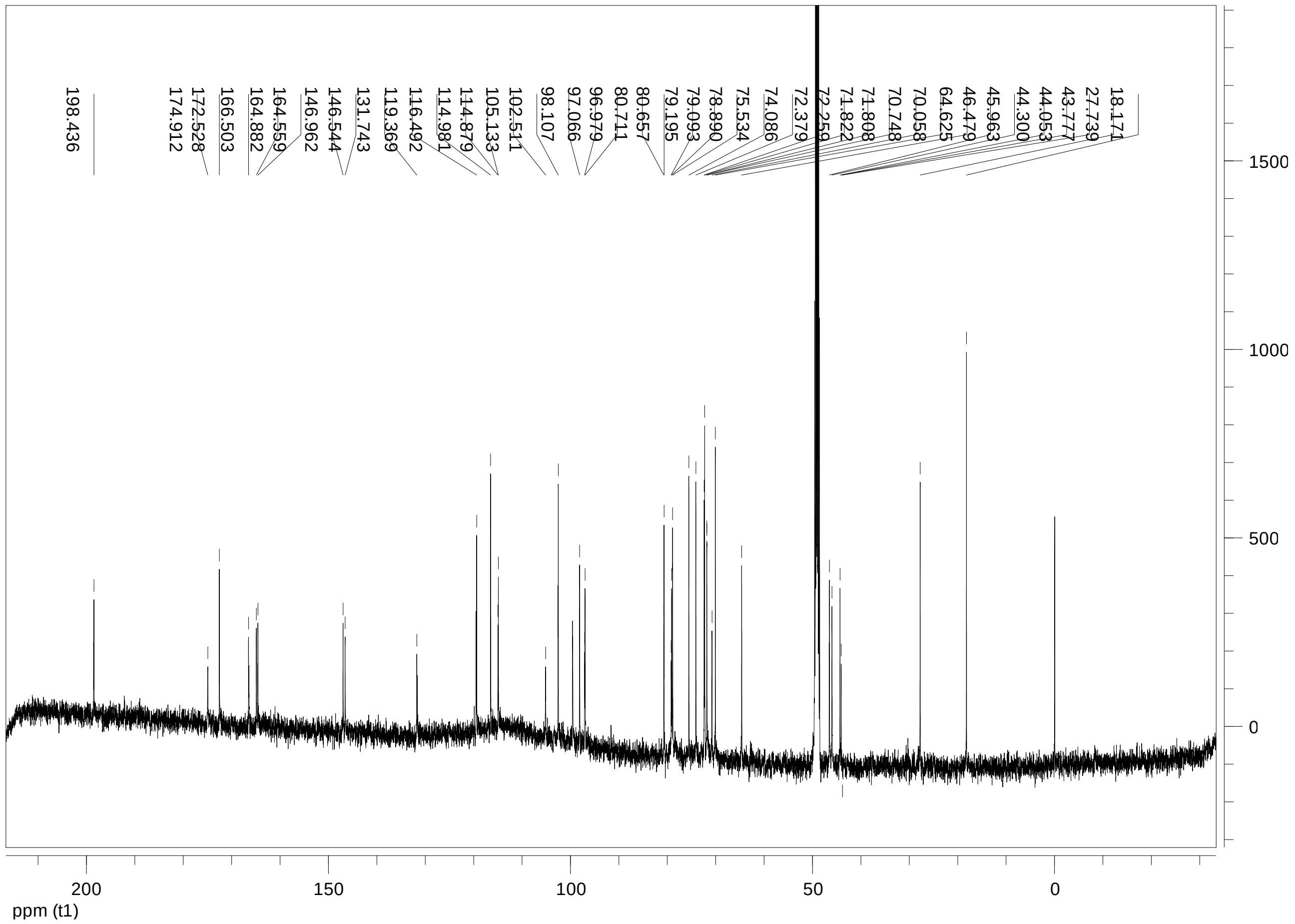

Figure S8. ^{13}C-NMR spectrum of pure Peripolin (**1**).

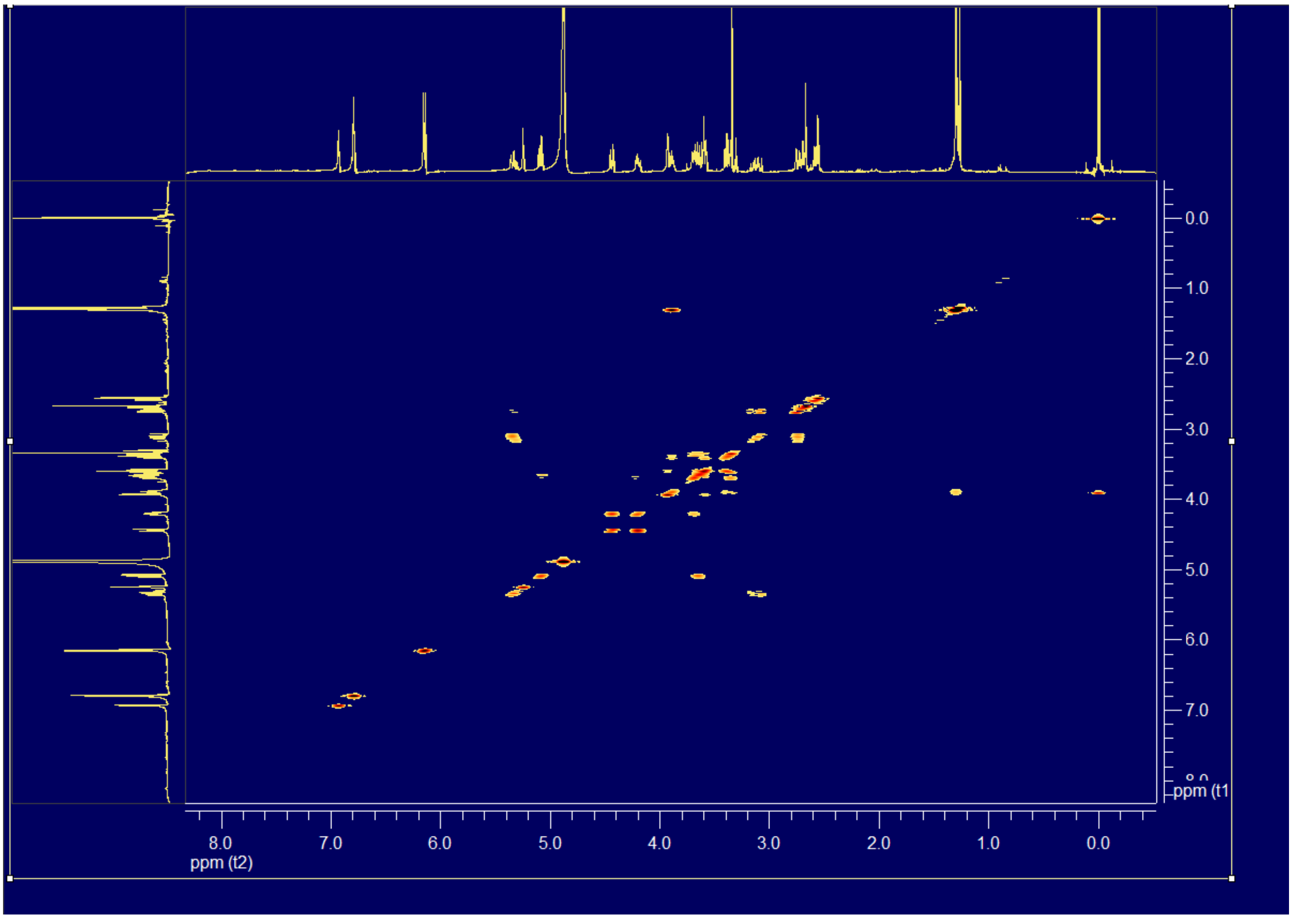

Figure S9. $^{1}H^{1}H$ COSY-NMR spectrum of pure Peripolin (**1**).

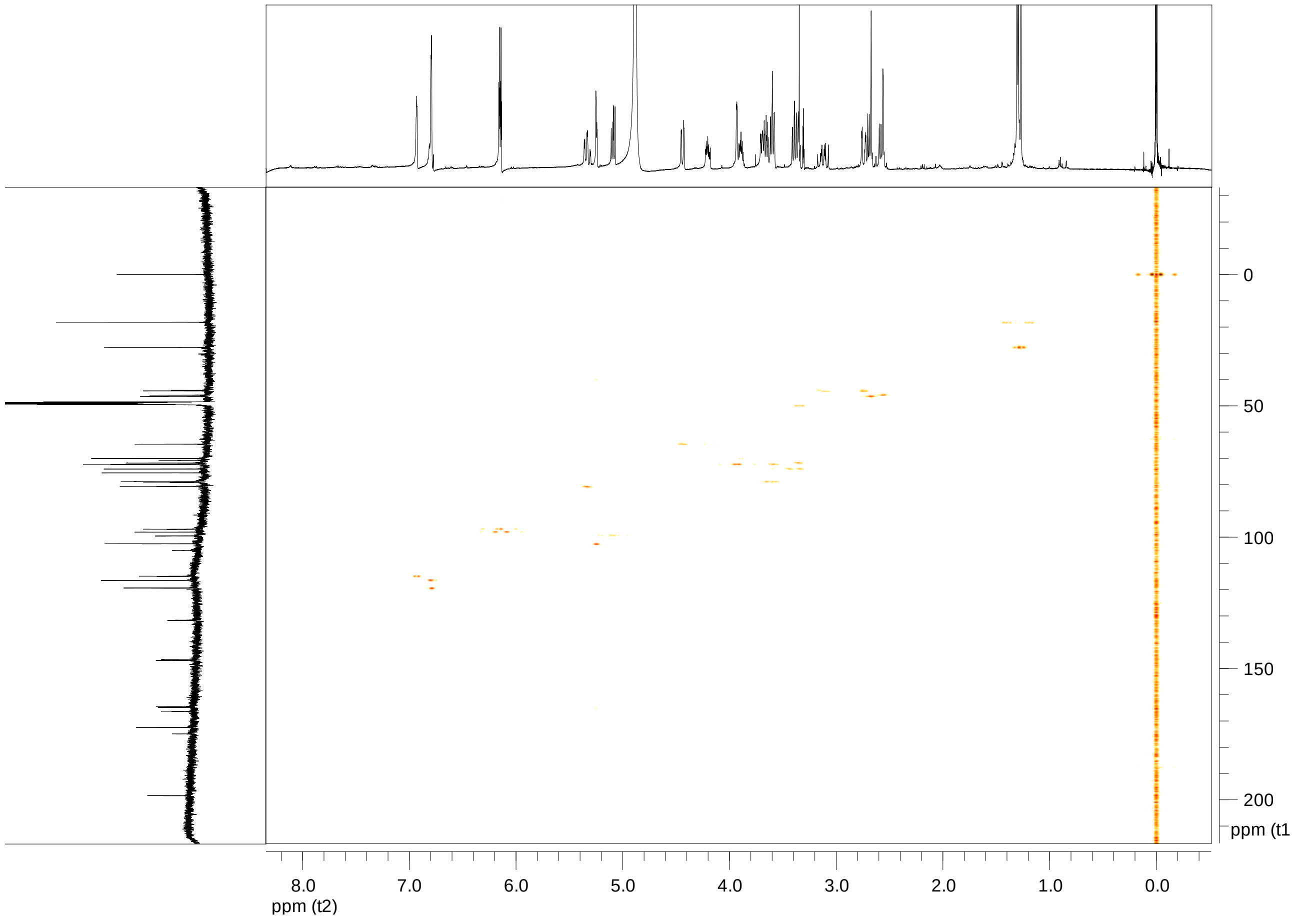

Figure S10. $^{1}H^{13}C$ HMQC-NMR spectrum of pure Peripolin (**1**).

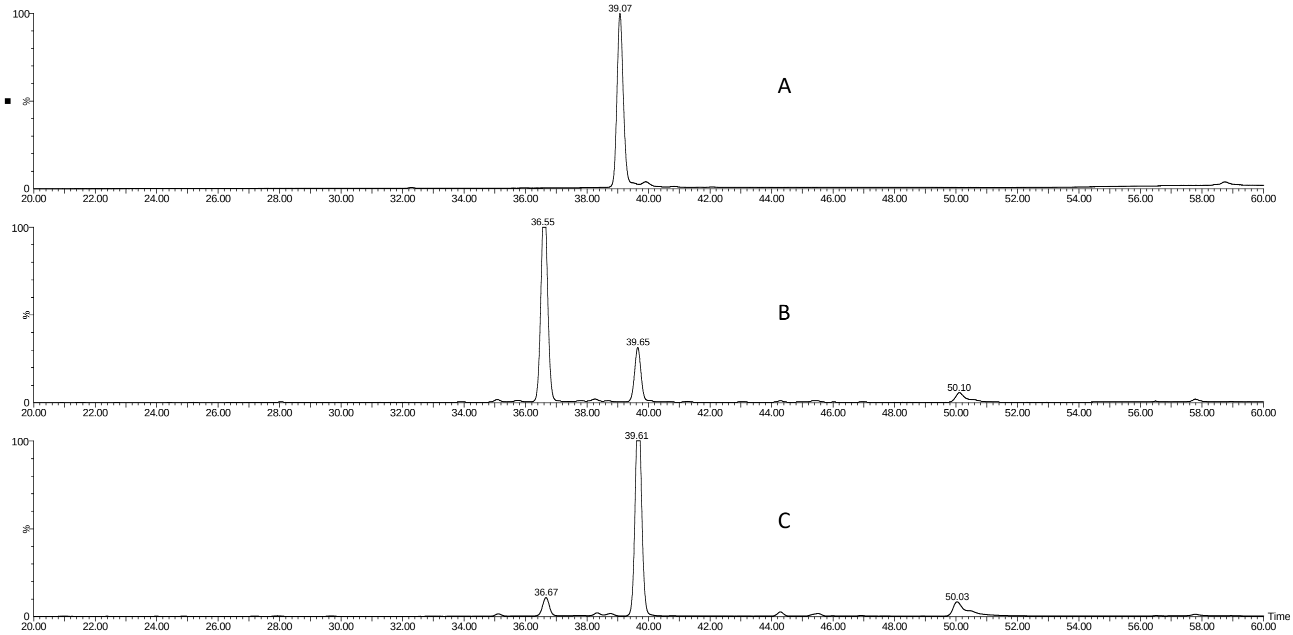

Figure S11. Enzyme (neohesperidase) cleavage of the sugar moiety of Peripolin (**1**): (A) Signal at retention time 39.07 represents Peripolin at t = 0h; (B) signals at retention time 36.55 and 39.65 represent, respectively, enzyme reaction products eriodictyol 7-O-(6″-(3‴-hydroxy-3‴-methylglutaryl)-β-glucoside and eriodictyol at t = 4h; (C) signals at retention time 39.61 represents eriodictyol at t = 20h

Table S1. IC_{50} values for flavonoids and inhibition of DPPH radical for neoeriocitrin, peripolin and Trolox at 10 µM.

	DPPH IC_{50} *(µmol/L)*	ABTS IC_{50} *(µmol/L)*	FRAP IC_{50} *(µmol/L)*
Neoeriocitrin	9	11	9
Naringin	>200	>20	>400
Neohesperidin	120	11	10
Peripolin	17	19	14
Melitidin	>200	>20	>100
Brutieridin	>200	18	20

	Flavononoid Concentration *(µmol/L)*	DPPH Inhibition *(%)*
Neoeriocitrin	10	58
Peripolin	10	27
Trolox	10	17

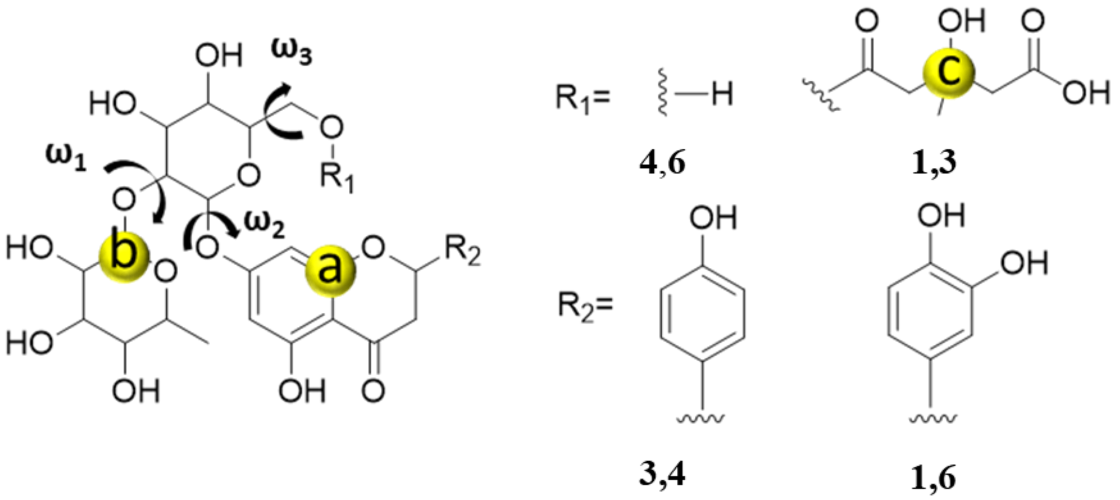

Scheme S3. Dihedral angles and center of masses investigated during the MD simulations of compounds **1**, **3**, **4** and **6**.

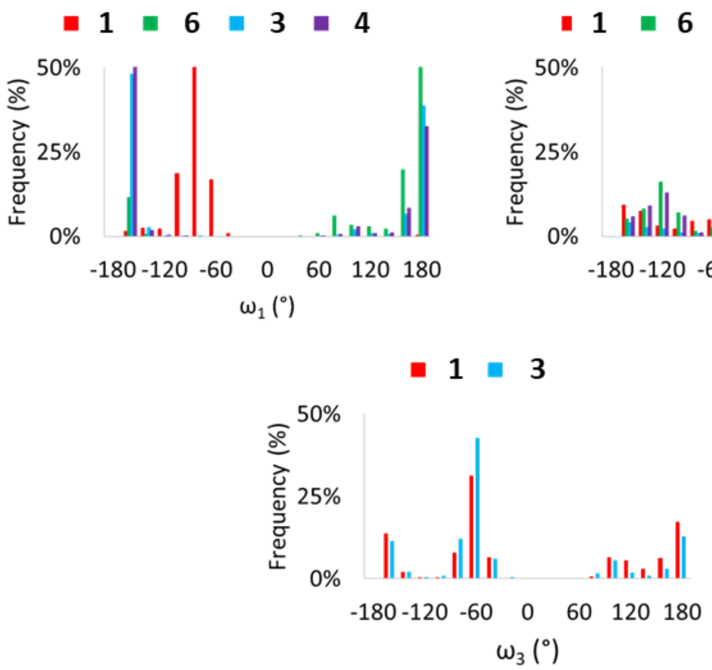

Figure S12. Frequency distribution of dihedral angle from MD trajectories.

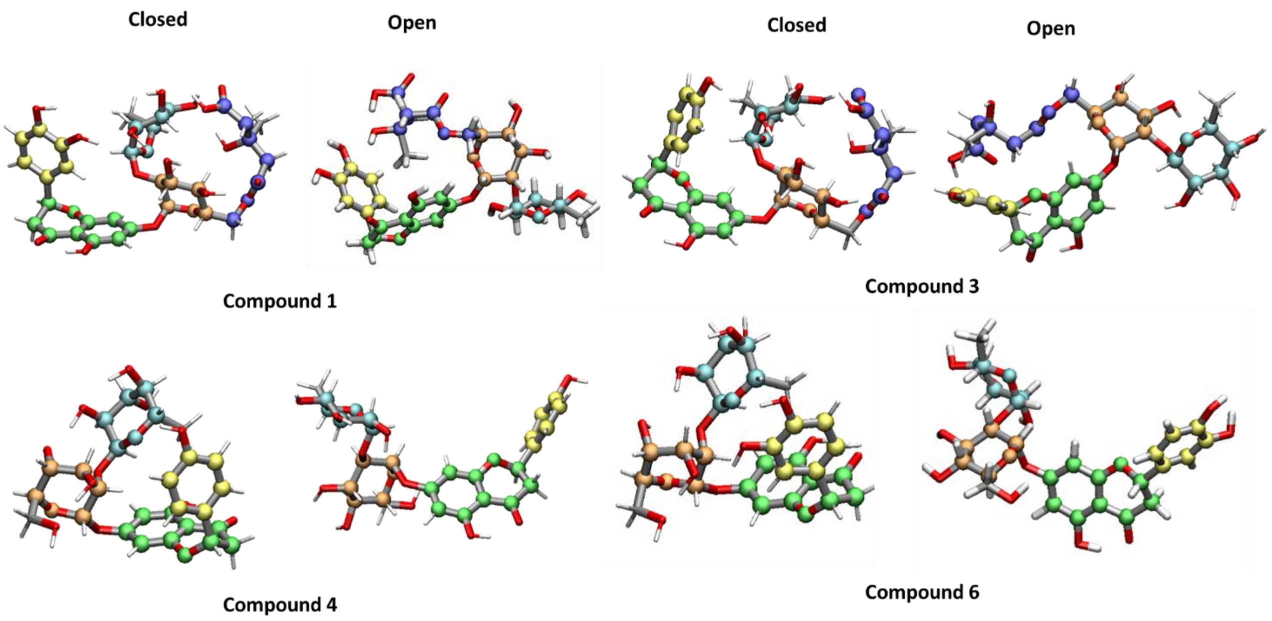

Figure S13. Optimized structures of compounds **1**, **3**, **4** and **6**, in both closed and open conformations, at PCM(ethanol)/B3LYP-D3/6-31+G(d,p) level of theory.

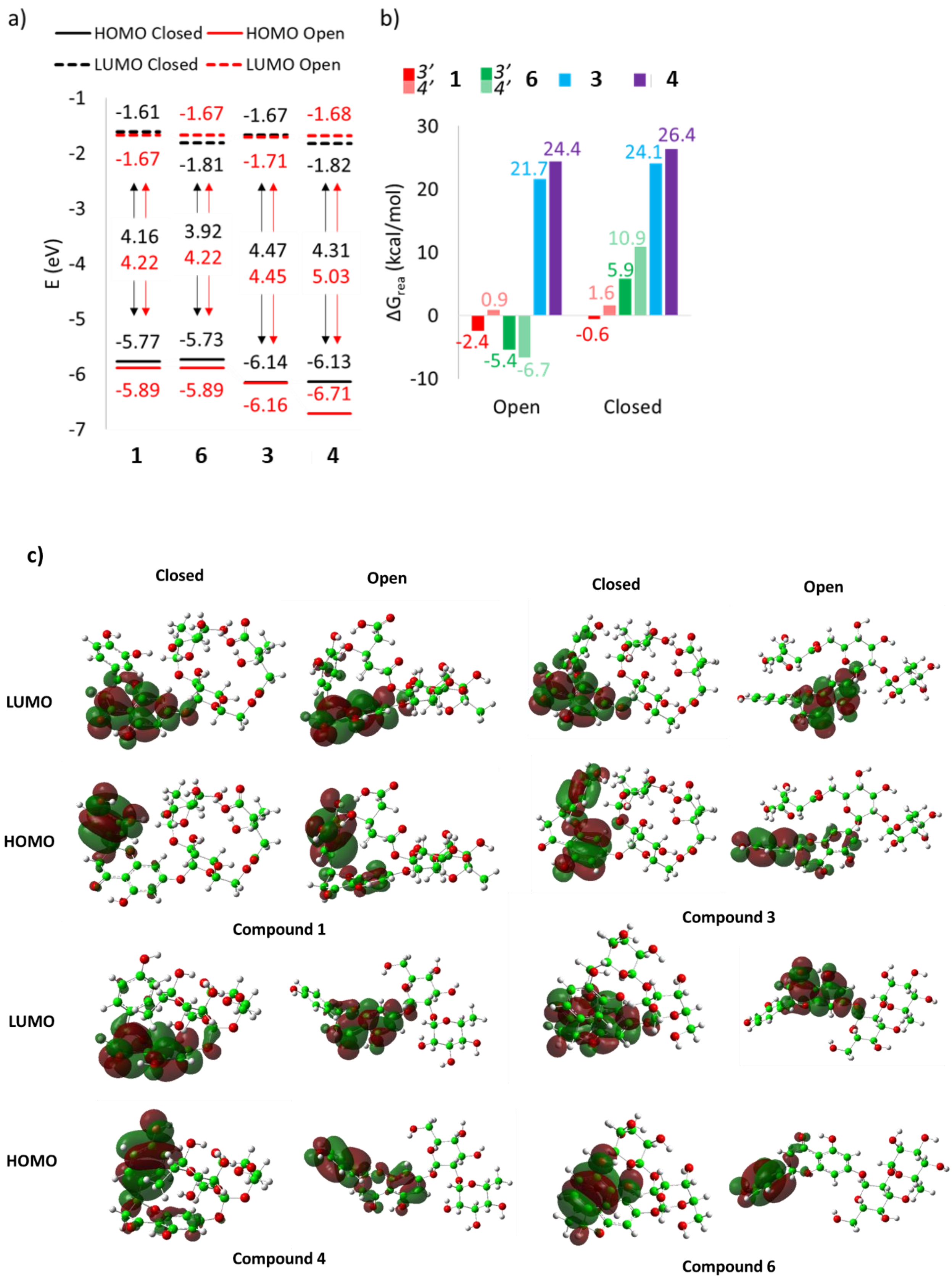

Figure S14. a) Calculated HOMO-LUMO gaps, b) ΔG_{rea} for **1**, **3**, **4** and **6** species c) HOMO-LUMO localization, in both open and closed conformations of compounds **1**, **3**, **4** and **6**.
